# Carbon-Coated Iron Oxide Nanoparticles Promote Reductive Stress-Mediated Cytotoxic Autophagy in Drug-Induced Senescent Breast Cancer Cells

**DOI:** 10.1021/acsami.3c17418

**Published:** 2024-03-14

**Authors:** Anna Lewińska, Adrian Radoń, Kacper Gil, Dominika Błoniarz, Agnieszka Ciuraszkiewicz, Jerzy Kubacki, Mariola Kądziołka-Gaweł, Dariusz Łukowiec, Piotr Gębara, Agnieszka Krogul-Sobczak, Piotr Piotrowski, Oktawia Fijałkowska, Sylwia Wybraniec, Tomasz Szmatoła, Aleksandra Kolano-Burian, Maciej Wnuk

**Affiliations:** † Institute of Biotechnology, College of Natural Sciences, University of Rzeszow, Pigonia 1, 35-310 Rzeszow, Poland; ‡ Łukasiewicz Research Network—Institute of Non-Ferrous Metals, Sowińskiego 5, 44-100 Gliwice, Poland; § Institute of Physics, Faculty of Science and Technology, University of Silesia in Katowice, 75 Pułku Piechoty 1, 41-500 Chorzów, Poland; ∥ Faculty of Mechanical Engineering, Silesian University of Technology, Konarskiego 18A, 44-100 Gliwice, Poland; ⊥ Department of Physics, Częstochowa University of Technology, Armii Krajowej 19, 42-200 Częstochowa, Poland; # Faculty of Chemistry, University of Warsaw, Pasteura 1, 02-093 Warsaw, Poland; ∇ Center of Experimental and Innovative Medicine, University of Agriculture in Krakow, Mickiewicza 24/28, 30-059 Krakow, Poland

**Keywords:** Fe_3_O_4_ nanoparticles, carbon coating, reductive stress, cytotoxicity, breast cancer, chemotherapy-induced senescence

## Abstract

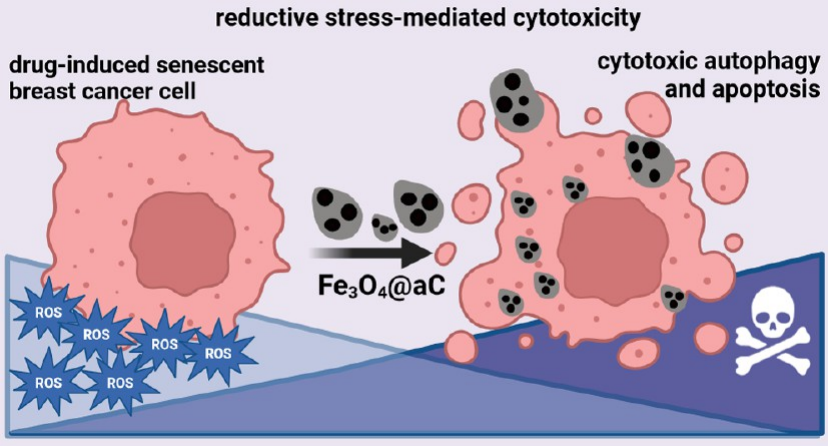

The surface modification of magnetite nanoparticles (Fe_3_O_4_ NPs) is a promising approach to obtaining biocompatible and multifunctional nanoplatforms with numerous applications in biomedicine, for example, to fight cancer. However, little is known about the effects of Fe_3_O_4_ NP-associated reductive stress against cancer cells, especially against chemotherapy-induced drug-resistant senescent cancer cells. In the present study, Fe_3_O_4_ NPs *in situ* coated by dextran (Fe_3_O_4_@Dex) and glucosamine-based amorphous carbon coating (Fe_3_O_4_@aC) with potent reductive activity were characterized and tested against drug-induced senescent breast cancer cells (Hs 578T, BT-20, MDA-MB-468, and MDA-MB-175-VII cells). Fe_3_O_4_@aC caused a decrease in reactive oxygen species (ROS) production and an increase in the levels of antioxidant proteins FOXO3a, SOD1, and GPX4 that was accompanied by elevated levels of cell cycle inhibitors (p21, p27, and p57), proinflammatory (NFκB, IL-6, and IL-8) and autophagic (BECN1, LC3B) markers, nucleolar stress, and subsequent apoptotic cell death in etoposide-stimulated senescent breast cancer cells. Fe_3_O_4_@aC also promoted reductive stress-mediated cytotoxicity in nonsenescent breast cancer cells. We postulate that Fe_3_O_4_ NPs, in addition to their well-established hyperthermia and oxidative stress-mediated anticancer effects, can also be considered, if modified using amorphous carbon coating with reductive activity, as stimulators of reductive stress and cytotoxic effects in both senescent and nonsenescent breast cancer cells with different gene mutation statuses.

## Introduction

Despite the increasing availability of targeted anticancer therapies and immunotherapies, chemotherapy is still commonly used to treat a number of cancers, for example, triple-negative breast cancer (TNBC).^1^ Unfortunately, drug resistance is widely observed due to anticancer drug treatment, thus limiting chemotherapeutic effects. Furthermore, drug resistance can be associated with the development of a chemotherapy-induced senescence program in cancer cells.^2,3^ Drug-induced senescence during chemotherapy may promote secondary senescence and malignant traits in neighboring cells by the action of increased secretion of proinflammatory factors, *i.e.*, senescence-associated secretory phenotype (SASP).^3,4^ Thus, numerous genetic and pharmacological approaches, such as nanobased approaches, have been developed to eliminate senescent cells by stimulating senolytic effects and inhibit secretory profiles of senescent cells by inducing senostatic effects.^5−7^

Surface-modified magnetic nanoparticles (NPs), such as magnetite (Fe_3_O_4_) NPs, in the form of multifunctional nanoplatforms and/or biocompatible core–shell nanostructures, are powerful theranostic tools with numerous biomedical applications, for example, bioimaging, biosensing, drug and gene delivery, and hyperthermia.^8−11^ However, uncontrolled release of ferric iron from Fe_3_O_4_ NPs may promote the generation of reactive oxygen species (ROS) and related toxicity in biological systems that can be considered as a double-edged sword with detrimental effects in normal cells and tissues and, if programmed, therapeutic effects against cancer cells and solid tumors.^12−15^ It is widely accepted that oxidative stress, a result of increased production of ROS and/or diminution in the antioxidant defense system (*e.g.*, decreased levels of antioxidants, decreased functionality of antioxidant transcription factors), may promote oxidative damage to biomolecules that may be associated with the initiation and progression of human pathologies, such as age-related diseases, namely, neurogenerative and cardiovascular disorders and cancer.^16^ The role of ROS in cancer biology is rather complex.^16^ Cancer cells, with high metabolic rates and a relatively more oxidized intracellular environment than normal cells, adapt to high levels of ROS by the activation of a plethora of protective antioxidant responses. However, ROS are also needed for tumor development, for example, cancer initiation, progression, migration, invasion, and metastasis.^16^ On the other hand, reductive stress can also be detrimental to cellular physiology, and reductive stress inducers can be considered as a novel anticancer approach.^17,18^ Reductive stress can be manifested as an increased ratio of antioxidants to pro-oxidants due to the accumulation of reducing equivalents such as NADH, NADPH, and GSH.^17^ Reductive stress can disturb the activity of redox-sensitive signaling pathways, protein disulfide bond formation in the endoplasmic reticulum (ER), and mitochondrial homeostasis and metabolism.^17^ Thus, similar to oxidative stress, reductive stress can also stimulate unfolded protein response (UPR)/ER stress and related cytotoxicity.^17,19^ However, more studies are needed to document the anticancer effects of reductive stress and underlying mechanisms and validate the nanosystems’ usefulness in reductive stress-mediated therapeutic action.

In the present study, we have designed, synthesized, and tested two core–shell-type modifications of Fe_3_O_4_ NPs against nonsenescent and drug-induced senescent breast cancer cells with different receptor and gene mutation statuses. As there are no data on nanoformulation-induced reductive stress-mediated anticancer effects, glucosamine-based amorphous carbon coating with reductive activity (Fe_3_O_4_@aC) was used to evaluate the cytotoxic potential of reductive stress in drug-resistant senescent breast cancer cells. Furthermore, the biocompatible polymer-based shell composed of dextran^20^ (Fe_3_O_4_@Dex) was also considered and used to understand better the mechanisms responsible for the observed reductive stress effect. The dextran-coated magnetite nanoparticles were tested in various biomedical applications, including drug delivery and MRI contrast production.^20,21^ Moreover, the coating by the dextran was confirmed as an efficient way to produce biocompatible magnetite nanoparticles.^22^ Despite the encapsulation of Fe_3_O_4_ NPs into polymers, only several studies describe the influence of the encapsulation of magnetite nanoparticles in carbon-based nanostructures. In the case of nanoparticles with a carbon layer, their toxic effects on cancer cells can be different and more complex, depending on the functional groups present in the carbon surface.^23,24^ Moreover, the chemical composition (doping) and number of presented on the surface functional groups are associated with the precursor and synthesis method used to prepare carbon-based material.^25−27^ As presented, Fe_3_O_4_@aC stimulated reductive stress in etoposide-induced senescent breast cancer cells that was accompanied by the inflammatory response, the activation of the autophagic pathway, and nucleolar stress leading to apoptotic cell death. The potential biomedical implications of the obtained results are discussed.

## Materials and Methods

### Synthesis of Fe_3_O_4_ NPs (Fe_3_O_4_@Dex and Fe_3_O_4_@aC)

Fe_3_O_4_ NPs encapsulated in dextran 70,000 and glucosamine-based amorphous carbon were synthesized using the polyol method. Briefly, 10 mmol Fe(acac)_3_ was dissolved in 100 mL of triethylene glycol (TREG). The organic modifier (dextran 70,000 or d-glucosamine sulfate potassium chloride) was dissolved in 50 mL of TREG and added into a Fe(acac)_3_ solution under continuous stirring. The solution was heated to 270 °C and the synthesis was then carried out for 1 h. Afterward, the mixture was cooled to room temperature, and 100 mL of ethyl acetate was added to precipitate Fe_3_O_4_ NPs and stirred for 10 min. The black precipitate was obtained by centrifugation (8000 rpm, 5 min) and washed thrice with ethyl acetate to remove TREG. Fe_3_O_4_@aC was purified from the inorganic salts (K_2_SO_3_ and K_2_Fe_2_(SO_4_)_3_), which spontaneously crystallized in the presence of KCl. Briefly, the sample was redispersed in DI water using ultrasonication and collected using the neodymium magnet 3 times. Afterward, the sample was washed with ethanol twice. Samples were dried at 60 °C for 12 h. Fe_3_O_4_ NPs synthesized in the presence of dextran 70,000 were denoted as Fe_3_O_4_@Dex and in the presence of d-glucosamine sulfate potassium chloride were marked as Fe_3_O_4_@aC (according to their chemical composition).

### Analysis of the Structure and Properties of Fe_3_O_4_ NPs

The phase purity of synthesized samples and the average crystallite size were determined based on the X-ray diffraction (XRD) method using a Rigaku MiniFlex 600 equipped with copper tube Cu Kα (λ = 0.15406 nm) as a radiation source. The data were analyzed using Rigaku Data Analysis Software PDXL2. The average crystallite size (*d*_XRD_) was calculated according to the Halder–Wagner method widely used for the determination of the crystallite size of nanoparticles.^28,29^ Fourier transform infrared (FTIR) spectra were collected for the samples and organic modifiers using the KBr pellet method in infrared transmission mode using a Nicolet 6700/8700 FTIR spectrometer. X-ray photoemission spectra in a wide −2 to 1400 eV binding energy range were measured using a PHI5700/660 Physical Electronics spectrometer (Al Kα monochromatic X-ray source with an energy of 1486.6 eV). ^57^Fe Mössbauer spectrometry using an MS96 Mössbauer spectrometer with a linear arrangement of a ^57^Co:Rh source was used as a complementary method for structural and magnetic properties analysis. The spectrometer was calibrated at room temperature with a 30-μm-thick α-Fe foil. Numerical analysis of the Mössbauer spectra was performed using MossWin4.0i software. Spectra were fitted with a hyperfine magnetic field (*B*_hf_) distribution. The model and implementation were based on the Voigt-based fitting method.^30^ Magnetic properties, *i.e.*, hysteresis loops and magnetization curves, were recorded at room temperature under the change of the magnetic field up to 20 kOe using a LakeShore VSM 7307 vibrating-sample magnetometer (VSM). The size distribution and average size of NPs were determined based on the transmission electron microscopy (TEM) image analysis. Briefly, the diameter of particles was measured for 100 different particles. The images (in TEM and scanning STEM modes) and selected area diffraction (SAED) patterns were obtained for nanoparticles redispersed in ultrapure ethanol and dropwise placed on a copper grid with a carbon film. All observations were performed using an S/TEM TITAN 80-300 transmission electron microscope. The hydrodynamic diameters and zeta-potential (ζ) were determined by a dynamic light scattering (DLS) instrument Zetasizer Nano-ZS (Malvern Instruments, U.K.). For the measurements of hydrodynamic diameters, a sample (1 mL) was placed in a polystyrene cuvette (10 × 10 × 45 mm^3^, cell type DTS0012), and data were collected at a 173° backscatter angle, using polarized laser light with a wavelength of 632.8 nm, at 25 °C, pH = 7.0, for the concentration range of samples from 0.25 to 1.00 mg/mL in water. Each result averages three measurements consisting of 11 runs for 10 s. For ζ-potential measurements, a sample was transferred to a U-shaped capillary cell with electrodes at each end (disposable folded capillary cell, DTS1070). The sample concentration was 0.25–1.00 mg/mL in water with a pH value of 7.0. Measurements were performed at 25 °C using an applied voltage of 150 V and polarized laser light with a wavelength of 632.8 nm. The Henry equation was applied to calculate the ζ-potential, using a viscosity of 0.8872 cP, a dielectric constant of 78.5, and a Henry function of 1.5. Thermogravimetric analysis was conducted under a nitrogen atmosphere using a TA Instruments Q50 Thermal Gravimetric Analyzer with a heating rate of 5 °C/min in the range of 50–750 °C. Analyzed samples were vacuum-dried at 40 °C before analysis. Finally, Raman spectra measurements were performed using a Renishaw’s Raman in Via Reflex spectrometer equipped with a Leica research-grade confocal microscope. Excitations were made by an ion-argon laser with a beam with a wavelength λ = 514 nm and with a plasma filter for 514 nm.

### Cell Lines and Culture Conditions

The following breast cancer cell lines were used: (a) triple-negative breast cancer cells (TNBC) MDA-MB-231 (HTB-26), MDA-MB-468 (HTB-132), Hs 578T (HTB-126), and BT-20 (HTB-19); (b) HER2-positive SK-BR-3 (HTB-30); and (c) ER-positive MCF-7 (HTB-22), HCC1500 (CRL-2329), and MDA-MB-175-VII (HTB-25) (ATCC, Manassas, VA). For selected experiments, normal human cells were also used, namely, a nontumorigenic epithelial cell line MCF 10F originated from the mammary gland (CRL-10318) and BJ skin fibroblasts (CRL-2522) (ATCC, Manassas, VA). Breast cancer cells and BJ cells were grown at 37 °C in a dedicated Dulbecco’s modified Eagle medium (DMEM) or a Roswell Park Memorial Institute (RPMI) 1640 medium with the addition of 10% (v/v) fetal bovine serum (FBS) and antibiotic/antimycotic mix (100 U/mL penicillin, 0.1 mg/mL streptomycin, and 0.25 μg/mL amphotericin B) (Corning, Tewksbury, MA) in a 5% CO_2_ incubator. MCF 10F cells were grown in a DMEM/Ham’s Nutrient Mixture F12 (51448C, Merck KGaA, Darmstadt, Germany) supplemented with 5% horse serum (H1270), 10 μg/mL human insulin (I9278), 10 ng/mL hEGF (E9644), 0.5 μg/mL hydrocortisone (H0888), 100 ng/mL cholera toxin from *Vibrio cholerae* (C8052) (Merck KGaA, Darmstadt, Germany), and an antibiotic/antimycotic mix (100 U/mL penicillin, 0.1 mg/mL streptomycin, and 0.25 μg/mL amphotericin B) (Corning, Tewksbury, MA). Cells were routinely passaged using a trypsin/EDTA solution (0.05% trypsin for MCF 10F cells, 0.25% trypsin for other cells, Corning, Tewksbury, MA) and seeded at a density of 10^4^ cells per cm^2^. For the evaluation of metabolic activity (MTT test), cells were treated with encapsulated NPs (Fe_3_O_4_@Dex and Fe_3_O_4_@aC) at concentrations of 1, 10, and 100 μg/mL for 4 and 24 h (96-well plate, 5 × 10^3^ and 10^4^ cells per a well). On the basis of MTT results, the concentration of 100 μg/mL and 4 h treatment were selected for further analysis.

### Uptake of Encapsulated NPs

Acridine orange staining-based evaluation of lysosomal activity^31^ was used to assess the uptake of Fe_3_O_4_@Dex and Fe_3_O_4_@aC. Briefly, upon stimulation with NPs, cells were washed and stained using a staining solution (1 μM acridine orange in Dulbecco’s phosphate-buffered saline, DPBS, Merck KGaA, Darmstadt, Germany) at 37 °C for 30 min. Lysosomal activity was analyzed using a confocal imaging system IN Cell Analyzer 6500 HS and IN Carta software (Cytiva, Marlborough, MA). The lysosomal activity was calculated according to the following formula: the number of lysosomes per cells/red channel fluorescence × lysosome area.

### Levels of Reactive Oxygen Species (ROS)

Upon stimulation with NPs, intracellular levels of total ROS (CellROX Green Reagent, C10444, Thermo Fisher Scientific, Waltham, MA) and mitochondrial superoxide (MitoSOX Red superoxide indicator, M36008, Thermo Fisher Scientific, Waltham, MA) were analyzed in live cells using a confocal imaging system IN Cell Analyzer 6500 HS and IN Carta software (Cytiva, Marlborough, MA). Data are presented as relative fluorescence units (RFU). Total superoxide levels were also evaluated using flow cytometry (Muse Cell Analyzer and the Muse Oxidative Stress Kit containing a superoxide indicator dihydroethidium, Luminex Corporation, Austin, TX).^32^ Representative histograms are shown. The Muse Oxidative Stress Kit was also used to assess the redox activity of NPs in a cell-free *in vitro* system, namely, dihydroethidium was added to a DPBS-based solution of NPs, and fluorescence was measured using a fluorescence mode microplate reader (λ_EX_ = 518, λ_EM_ = 605). Data were normalized to fluorescent signals of dihydroethidium added to DPBS.

### Analysis of the Mode of Cell Death (Apoptosis versus Necrosis)

Upon stimulation with NPs, apoptotic and necrotic cell death was studied using flow cytometry and Annexin V and 7-AAD dual staining (Muse Cell Analyzer and the Muse Annexin V and Dead Cell Assay Kit, Luminex Corporation, Austin, TX).^32^ Four subpopulations were revealed: live (dual staining-negative), early apoptotic (Annexin V-positive), late apoptotic (dual staining-positive), and necrotic cells (7-AAD-positive). Data are presented as % and representative dot plots.

### Activation of the Senescence Program and the Analysis of Senescence-Associated-β-Galactosidase (SA-βGAL) Activity

To stimulate chemotherapy-induced senescence, 24 h treatment with 1 μM etoposide (Merck KGaA, Darmstadt, Germany) was used. The drug was then removed, and cells were cultured for up to 10 days to activate the senescence program. The cell culture medium was changed every 48 h to avoid a starvation effect. Imaging cytometry and the CellEvent Senescence Green Detection Kit were used to analyze SA-βGAL activity as comprehensively described elsewhere.^33^ SA-βGAL activity is presented in relative fluorescence units (RFU).

### Imaging Cytometry

Upon stimulation with NPs, fixation and the immunostaining protocol were used as previously described.^33^ The following primary and secondary antibodies were used: anticaspase 3 (1:500, PA5-77887), anticaspase 9 (1:100, PA5-17913), anti-p21 (1:800, MA5-14949), anti-p27 (1:500, PA5-27188), anti-p53 (1:100, MA5-12557), anti-p57 (1:100, PA5-82042), anti-FOXO3a (1:200, MA5-14932), anti-SOD1 (1:200, PA1-30195), anti-SOD2 (1:500, MA1-106), anti-GPX4 (1:100, PA5-120674), anti-ACSL4 (1:250, PA5-100033), anti-LC3B (1:500, PA5-32254), anti-BECN1 (1:100, TA502527), antitransferrin receptor (TfR) (1:250, 13-6890), antiferritin (1:250, MiF2502), anti-NFκB p65 (1:100, PA5-16545), anti-IL-6 (1:100, TA500067), anti-IL-8 (1:500, M801), anti-RRN3 (1:100, PA5-30872), antinucleolar antigen (1:100, MA1-91240), anti-NSUN1 (NOP2) (1:250, PA5-59073), antilamin A/C (1:100, MA3-1000), antilamin B1 (1:500, PA5-19468), anti-53BP1 (1:100, PA5-17578), antirabbit IgG conjugated to Texas Red (TR) (1:1000, T2767), and antimouse IgG conjugated to FITC (1:1000, F2761). Nuclei were stained using Hoechst 33342 staining. To capture digital cell images and quantitative analysis of protein levels, the confocal imaging system IN Cell Analyzer 6500 HS and IN Carta software were used (Cytiva, Marlborough, MA). Protein levels (cytoplasmic or nuclear pools) are presented in relative fluorescence units (RFU). For the analysis of 53BP1, 53BP1 foci per nucleus were scored.

### Analysis of Cell Cycle, Stress Responses, and Autophagy-Related Gene Mutations

Data on the gene mutation status in breast cancer cell lines used in this study were obtained from the Dependency Map (DepMap) portal (https://depmap.org/portal/). Gene enrichment analysis was based on three databases, namely, Reactome, KEGG, and GO Pathways, using Kobas standalone software.^34^ Pathways with a corrected P-value under 0.05 (FDR correction method of Benjamini and Hochberg^35^) were chosen for further analysis using UpSetR software.^36^

### Statistical Analysis

The results were calculated on the basis of the mean ± standard deviation from three independent replicates. Box and whisker plots with median, lowest, and highest values were also applied. Differences between untreated conditions and the treatment with encapsulated iron oxide NPs were studied using one-way analysis of variance (ANOVA) and Dunnett’s multiple comparison test (GraphPad Prism 5). Furthermore, differences between Fe_3_O_4_@Dex and Fe_3_O_4_@aC treatments were analyzed using one-way ANOVA and Tukey’s multiple comparison test (GraphPad Prism 5). *P*-values of less than 0.05 were assumed as significant.

## Results and Discussion

### Structure and Magnetic Properties of Fe_3_O_4_ NPs

**Figure 1:**
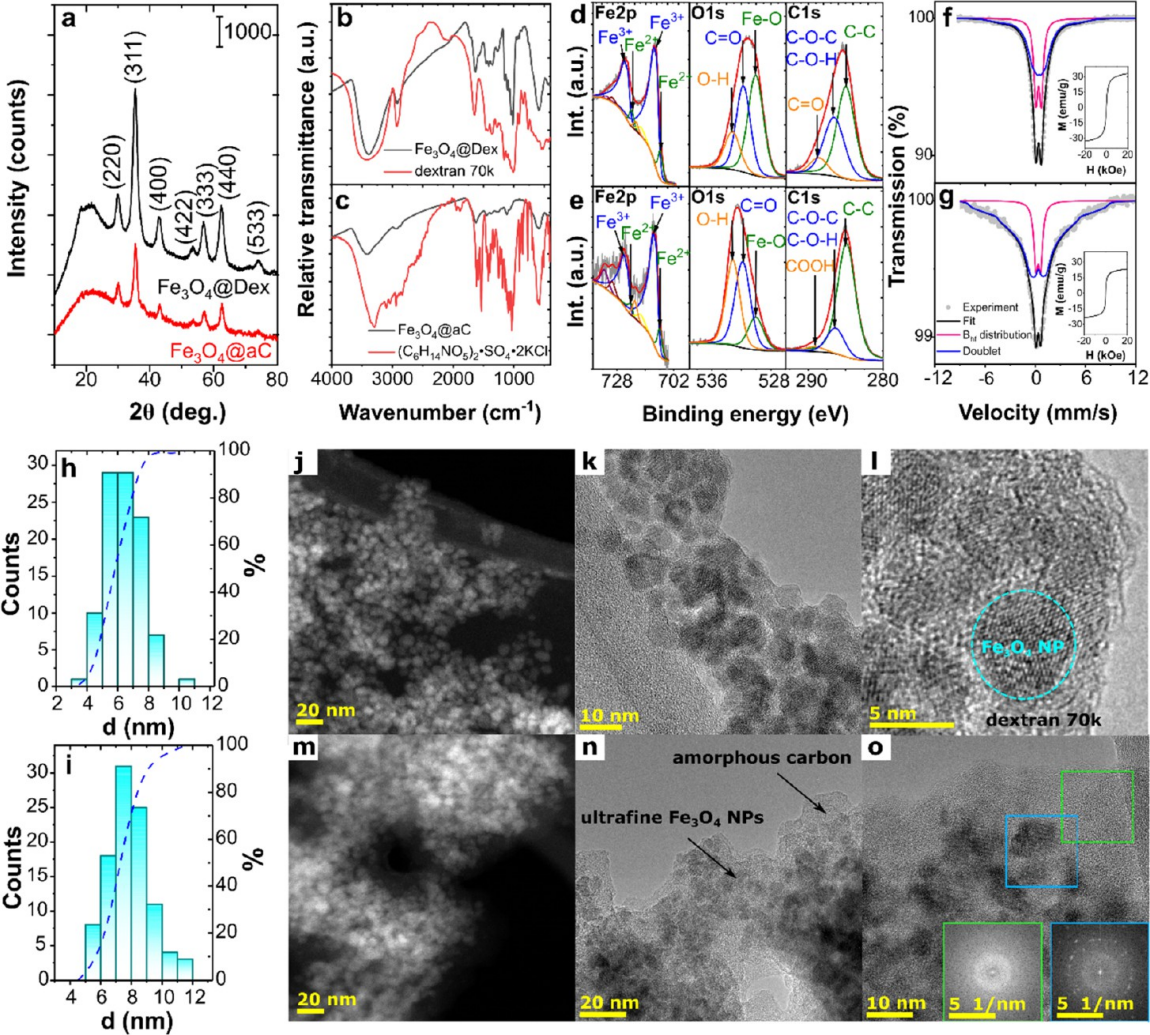
Structure and magnetic properties of Fe_3_O_4_@Dex and Fe_3_O_4_@aC: XRD patterns of samples (a) with marked Miller indices of the magnetite spinel phase; comparison of FTIR spectra of Fe_3_O_4_@Dex (b) and Fe_3_O_4_@aC (c) with spectra of pure organic modifiers; deconvolution of XPS spectra of Fe 2p, O 1s, and C 1s core lines obtained for Fe_3_O_4_@Dex (d) and Fe_3_O_4_@aC (e); and Mössbauer spectra of Fe_3_O_4_@Dex (f) and Fe_3_O_4_@aC (g) with fitted curves (the dots, black solid lines, pink and blue represent experimental data, fitted curves, hyperfine magnetic distribution and doublets, respectively). Insets show the recorded VSM curves; size-distribution histograms with cumulative curves plotted for Fe_3_O_4_@Dex (h) and Fe_3_O_4_@aC (i) and TEM images of Fe_3_O_4_@Dex (j–l) and Fe_3_O_4_@aC (m–o) nanoparticles in STEM (j, m) and HRTEM (k, l, n and o) modes; insets show the FFTs from the green and blue marked areas (o).

The structure and morphology of synthesized Fe_3_O_4_@Dex and Fe_3_O_4_@aC were determined by analyzing XRD patterns and TEM images. As shown in Figure 1a, both samples are characterized by spinel-phase cubic structure AB_2_O_4_ described in the *Fd*3̅*m* space group without visible any additional diffraction peaks related to the presence of crystalline impurities. However, the second amorphous phase was also noted for both samples (broad diffraction halo primarily visible between 10 and 30°). While the phase composition of both samples is similar, the difference was observed under the average crystallite size (*d*_XRD_) analysis. The *d*_XRD_ value (Figure S1a) of Fe_3_O_4_@Dex (4.70 ± 0.12 nm) was nearly 2 times lower than Fe_3_O_4_@aC (7.56 nm ±0.72) and confirmed the differences between the crystallization process in the presence of dextran 70,000 and d-glucosamine sulfate potassium chloride.

This crystallization process is related to encapsulating the magnetite nanoparticles in a polymer or an amorphous carbon shell. The presence of these shells was first confirmed by FTIR spectroscopy. As shown in Figure 1b, FTIR spectra of Fe_3_O_4_@Dex and pure dextran 70,000 are very similar. The two vibrations observed for Fe_3_O_4_@Dex at 591 and 440 cm^–1^ are characteristic of the Fe–O vibration in a magnetite crystal structure,^37^ while other vibrations confirm the presence of the polymer in the sample. The broad band at around 3400 cm^–1^ is characteristic of the hydroxyl groups and adsorbed water O–H stretching vibration. The other bands characteristic for polysaccharides were also observed for magnetite nanoparticles and pure dextran, including C–H stretching (∼2925 cm^–1^), C–O stretching (∼1415 cm^–1^), and stretching bands of C=C bonds from aromatic rings (∼1643 cm^–1^).^38^ The sharp peak related to the C–H stretching vibration related to the flexibility in the chain around the glycosidic bond at 1015 cm^–1^ and an additional one associated with the bending vibration at 764 cm^–1^ were observed in pure dextran and modified magnetite NPs. Also, the α-glycosidic bond appeared at 918 and 846 cm^–1^, and the glycosidic bond and C–O–C stretching vibration were observed at 1154 cm^–1^.^39,40^

In contrast, d-glucosamine and Fe_3_O_4_@aC FTIR spectra are different because, in the case of Fe_3_O_4_@aC, d-glucosamine degradation was noted (Figure 1c). The typical d-glucosamine bands disappeared during the magnetite crystallization process, which was related to the thermal decomposition of this organic compound and the formation of amorphous carbon containing a high concentration of functional groups, *i.e.*, a structure similar to the hydrochar (also see Raman spectra analysis presented in Figure S1b). Furthermore, the degradation process can also be confirmed by the analysis of energy-dispersive X-ray (EDX) spectra (Figure S1c), in which a sharp peak from the carbon can be noted only for the Fe_3_O_4_@aC sample. In the FTIR spectra of Fe_3_O_4_@aC, characteristic vibrations from the oxygen-rich functional groups’ amorphous carbon structure were observed, while the Fe–O bands were visible at 585 and 445 cm^–1^ (Figure 1c). The band at around 1624 cm^–1^ and the broad one with a maximum of 3423 cm^–1^ are related to the presence of water, while the band at around 1624 cm^–1^ can also be attributed to the presence of C=C bonds. Moreover, the shoulder visible at around 1715 cm^–1^ is associated with C=O mode characteristic for the COOH and C=O vibrations. Additionally, bands at 1379 and 1115 cm^–1^ are related to the C–OH vibration and at 1070 cm^–1^ to epoxy groups. Also, CH_*x*_ presence can be confirmed according to the presence of bands at around 1450 and 2922 cm^–1^.^41,42^ The presence of these oxygen-rich functional groups was confirmed by X-ray photoelectron spectroscopy (XPS) analysis. The XPS survey spectra of Fe_3_O_4_@Dex and Fe_3_O_4_@aC (Figure S1d,e, respectively) confirm the presence of C, O, and Fe in both samples and the presence of N in Fe_3_O_4_@aC. The presence of nitrogen was associated with doping of the obtained carbonaceous structure and was observed recently in the hydrochar structure synthesized from glucosamine hydrochloride.^43^ Complementary to the TEM and FTIR analyses, it can be seen that the carbon-based structure covers the Fe_3_O_4_@aC sample; therefore, the signal from the iron ions is weak.

Additionally, the presence of these oxygen-rich functional groups was confirmed using the thermogravimetric analysis (TGA) method. Thermogravimetric analysis of both samples revealed two-stage decomposition (Figure S2a,b). Around a 2% weight loss is observed up to around 130 °C, which can be attributed to removing the water that remains in the sample. Significant decomposition starts from 150 °C up to 500 °C for Fe_3_O_4_@aC and 420 °C for Fe_3_O_4_@Dex, with the total weight loss of 58 and 21%, respectively. This process is attributed to decomposition of the functional groups and confirms the high content of the carbon-based structure in Fe_3_O_4_@aC observed in the EDX and XPS spectra. Also, analysis of XPS spectra of Fe 2p, O 1s, and C 1s core lines (Figure 1d,e) confirms the presence on the magnetite surface polymeric and carbon-based shells. The observed Fe 2p core line is characteristic of magnetite and confirms the presence of Fe^3+^ and Fe^2+^ ions in both samples. The O 1s line is composed of components related to the presence of oxygen in hydroxyl and carbonyl (carboxyl) functional groups and the Fe_3_O_4_ structure. For Fe_3_O_4_@Dex, the C 1s line is much broader than for the Fe_3_O_4_@aC sample and is associated with the presence of C–C/C–H (285.0 eV), C–O–C/C–O–H (286.6 eV), and C=O (288.6 eV). Similar bonds (C–C/C–H at 284.9 eV, C–O–C/C–O–H at 286.3 eV, and O–C=O at 289.1 eV) were observed for the Fe_3_O_4_@aC sample.

Interestingly, the crystallization process in the presence of dextran 70,000 and d-glucosamine sulfate potassium chloride also changes the magnetic properties, confirmed by the analysis of Mössbauer spectra (MS) and hysteresis loops. The MS received at room temperature and hyperfine magnetic field distribution related to appropriate components are shown in Figures 1f,g and S1f,g, respectively. Hyperfine parameters for all fitted components are listed in Table 1. Spectra were made using one distribution of hyperfine fields and one doublet. The obtained spectra confirm the existence of superparamagnetic nanoparticles in analyzed samples. The doublet in the central part of the spectra represents the superparamagnetic particles.^44,45^ Both the doublet and the component related to the distribution of hyperfine fields are associated with the relaxation time distribution of the magnetic moments of iron atoms. In the superparamagnetic state, the direction of the magnetization of nanoparticles fluctuates among the easy axis of magnetization.

**Table 1:** Mössbauer Hyperfine Parameters Obtained for Fitted Components^a^

sample	IS (mm/s)	QS (mm/s)	*G* (mm/s)	*B*_hf_ (T)	*A* (%)	component
Fe_3_O_4_@Dex	0.43(1)	0.00(1)	0.55(2)	⟨11.3⟩	61.0	magnetic relaxation
	0.37(1)	0.66(1)			39.0	superparamagnetic
Fe_3_O_4_@aC	0.38(2)	0.03(2)	0.55(2)	⟨20.7⟩	82.7	magnetic relaxation
	0.35(1)	0.56(1)			17.3	superparamagnetic

a Where IS—isomer shift, QS—quadrupole splitting, G—full line width at half-maximum, and *B*_hf_—hyperfine magnetic field.

The relaxation time is the average time required to change the particle magnetization from one direction to another.^46^ The superparamagnetic nature of Fe_3_O_4_@Dex and Fe_3_O_4_@aC also confirms VSM curve analysis (insets are shown in Figure 1f,g, respectively). Fe_3_O_4_@Dex has saturation magnetization (*M*_s_) much higher (32.87 emu/g) than the sample encapsulated in amorphous carbon (23.58 emu/g), which is related to the higher content of the nonmagnetic phase in Fe_3_O_4_@aC. However, in both samples, we observed the same retentivity of about 0.01 emu/g and a low coercivity (*H*_c_) of 0.52 and 0.27 Oe for Fe_3_O_4_@Dex and Fe_3_O_4_@aC, respectively. The observed low coercivity values and retentivity close to 0 are related to the encapsulation of nanoparticles in polymeric and carbon-based matrices, which prevent the formation of a collective state and increase of the *H*_c_ value.^47^

As mentioned, the observed changes in the magnetic properties can be attributed to the presence of different shells but also to variations in particle size. Accordingly, particle size distribution and average size were determined based on the TEM images analysis and presented in Figure 1h,i for Fe_3_O_4_@Dex and Fe_3_O_4_@aC, respectively. As can be observed, the average particle size (*d*_av_) is equal to 6.33 ± 0.40 nm for particles with a dextran 70,000 shell and 7.85 ± 0.45 nm for particles encapsulated in amorphous carbon. Only a slight deviation was observed between *d*_XRD_ and *d*_av_ values for the Fe_3_O_4_@Dex sample and nearly the same value for Fe_3_O_4_@aC. While the average particle size differs for both samples, their morphology is similar. According to TEM images, the spherical-shaped nanoparticles were synthesized using both modifiers. The spinel-phase characteristic for Fe_3_O_4_ was additionally confirmed by analysis of SAED patterns (see Figure S1h,i). The presence of the amorphous carbon can be easily observed in both STEM (Figure 1m) and high-resolution TEM (HRTEM) (Figure 1n,o) images, while the presence of the dextran can be primarily visible in HRTEM images (Figure 1j–l). In both cases, nanoparticles are dispersed in the amorphous-like matrix. The coexistence of these two phases was additionally confirmed for the Fe_3_O_4_@aC sample using the fast Fourier transform (FFT) method. FFTs reveal (Figure 1o) that Fe_3_O_4_ NPs are embedded in the carbon phase, while magnetite nanoparticles in the Fe_3_O_4_@Dex sample are only slightly coated by the polymer matrix and form a noninteracting system, *i.e.*, each particle is coated by dextran 70,000.

The coating of the particles by both amorphous carbon and dextran 70,000 also influences aggregates’ dispersion stability. DLS measurements show the hydrodynamic diameters between 45 and 220 nm for Fe_3_O_4_@Dex particles and between *ca*. 400 and 3000 nm for Fe_3_O_4_@aC particles (Figure S3); thus, the analyzed samples are polydisperse. Although both samples are polydisperse, the size distribution is lower for Fe_3_O_4_@Dex.

For Fe_3_O_4_@Dex, ζ-potential is negative and changes from ζ = −22.4 ± 7.2 to ζ = −30.7 ± 6.9 mV (Figure S4a) as the concentration decreases from 1.00 to 0.25 mg/mL, whereas ζ for Fe_3_O_4_@aC is almost unchanged, *i.e.*, 16.5 ± 7.4, 16.8 ± 3.4, and 13.6 ± 3.6 for 1.00, 0.50, and 0.25 mg/mL, respectively (Figure S4b). Each of the presented values of ζ is an average obtained for all charged species present in the analyzed sample (for the polydispersed samples, as it is in the case of Fe_3_O_4_@Dex and Fe_3_O_4_@aC, the ζ-potential is reported as an average across all charged species). According to the literature, nanoparticles with a ζ-potential between −10 and +10 mV are considered approximately neutral, while nanoparticles with ζ-potentials greater than +30 mV or less than −30 mV are considered strongly cationic and strongly anionic, respectively. Thus, Fe_3_O_4_@Dex and Fe_3_O_4_@aC can be considered slightly anionic and cationic, respectively. Moreover, the dispersion stability of nanoparticles can be defined by the magnitude of ζ-potential, which indicates lower than moderate stability for both kinds of NPs, with Fe_3_O_4_@Dex particles being more stable than Fe_3_O_4_@aC. The changes in the stability and the ζ-potential value are associated with forming of two different shells on the magnetite surface.

### Carbon-Coated Fe_3_O_4_ NPs Induce Apoptosis and Necrosis in Nonsenescent Breast Cancer Cells That is Accompanied by Reduced Levels of ROS

**Figure 2:**
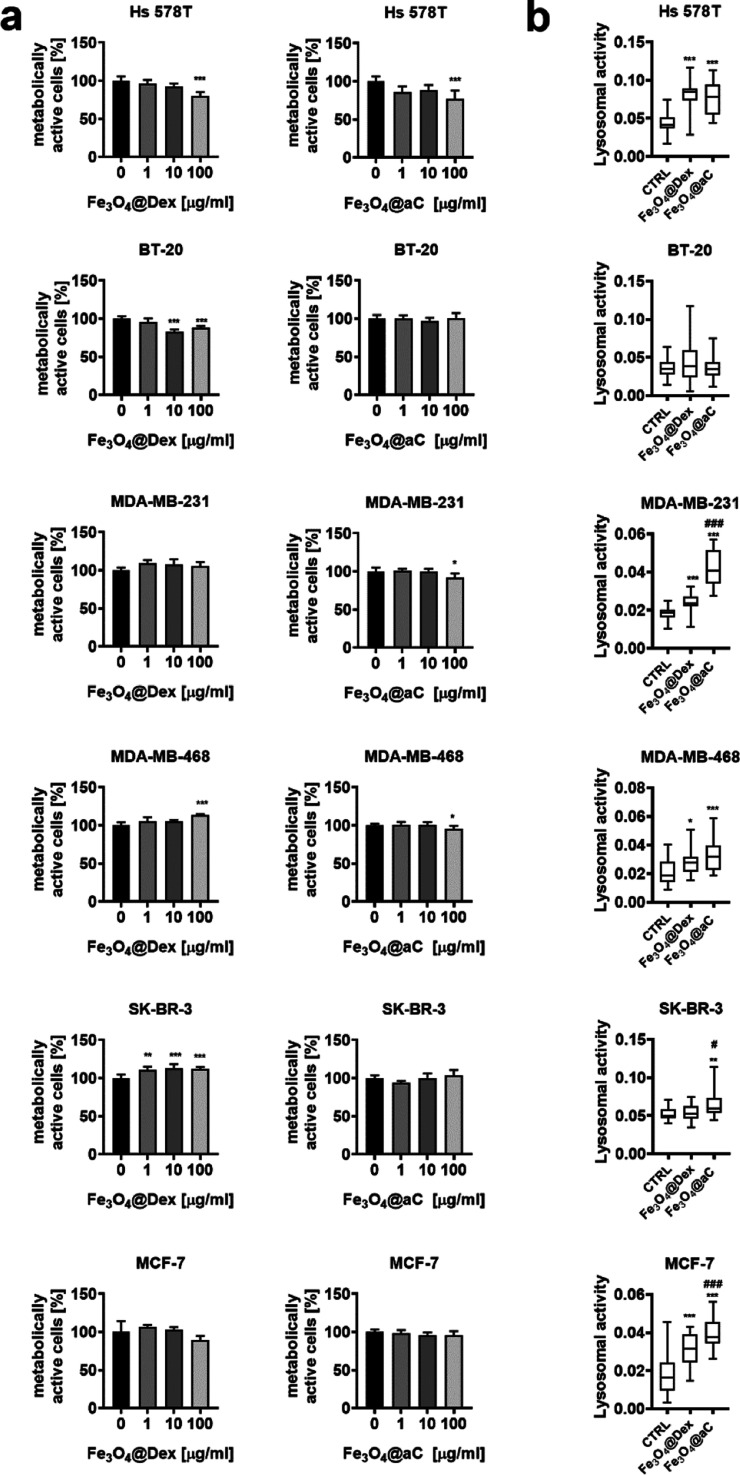

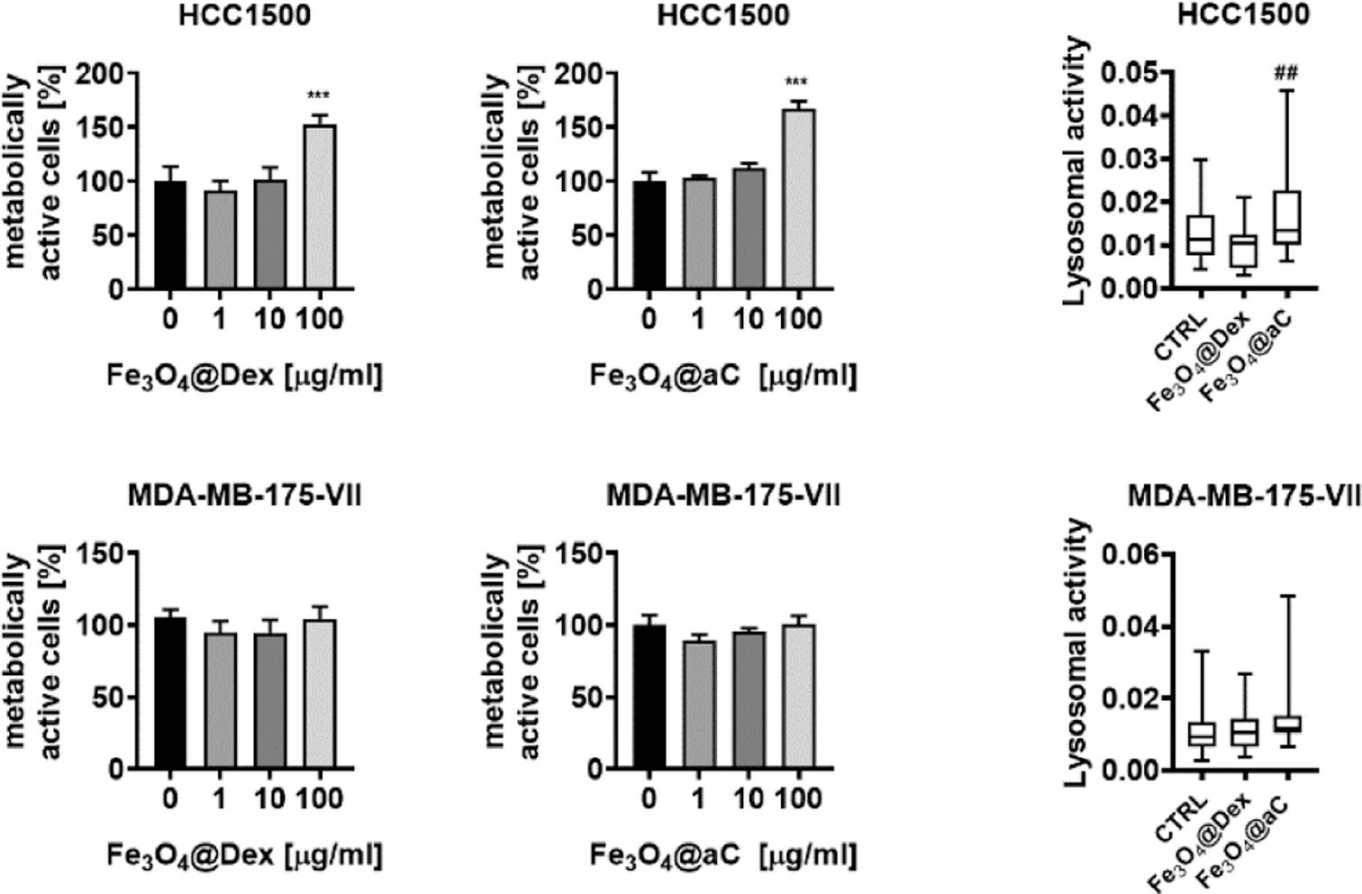
Effects of encapsulated Fe_3_O_4_ NPs (Fe_3_O_4_@Dex and Fe_3_O_4_@aC) on metabolic activity (a) and lysosomal activity (b) in breast cancer cells with different gene mutation statuses. (a) Cells (10,000 cells per well) were treated with 1, 10, and 100 μg/mL NPs for 4 h. Metabolic activity was assayed using the MTT test. Metabolic activity at standard growth conditions is considered as 100%. Bars indicate SD, *n* = 3, ***p < 0.001, **p < 0.01, and *p < 0.05 compared to untreated control (ANOVA and Dunnett’s a posteriori test). (b) Uptake of NPs was assessed as changes in lysosomal activity. Cells were treated with 100 μg/mL NPs for 4 h, and lysosomal activity was revealed using acridine orange staining and imaging cytometry. The lysosomal activity was calculated according to the formula: the number of lysosomes per cells/red channel fluorescence × lysosome area. Box and whisker plots are shown, *n* = 3, ***p < 0.001, **p < 0.01, and *p < 0.05 compared to untreated control (ANOVA and Dunnett’s a posteriori test); ^###^*p* < 0.001, ^##^*p* < 0.01, and ^#^*p* < 0.05 compared to Fe_3_O_4_@Dex treatment (ANOVA and Tukey’s a posteriori test). CTRL, untreated control; Fe_3_O_4_@Dex, dextran-based coated iron oxide nanoparticles; and Fe_3_O_4_@aC, glucosamine-based amorphous carbon-coated iron oxide nanoparticles.

We tested then the action of encapsulated Fe_3_O_4_ NPs, namely, dextran-based (Fe_3_O_4_@Dex) and glucosamine-based amorphous carbon-coated (Fe_3_O_4_@aC) NPs against eight phenotypically different breast cancer cells (triple-negative breast cancer cells (TNBC)—Hs 578T, BT-20, MDA-MB-231, and MDA-MB-468; estrogen receptor-positive breast cancer cells—MCF-7, HCC1500, and MDA-MB-175-VII; and HER2-positive breast cancer cells— SK-BR-3) using the analysis of metabolic activity (MTT assay) (Figures 2a and S5a). Breast cancer cells (5000 and 10,000 cells per well) were stimulated with Fe_3_O_4_@Dex and Fe_3_O_4_@aC at concentrations of 1, 10, and 100 μg/mL for 4 h (Figures 2a and S5a). In general, 1 and 10 μg/mL concentrations did not affect the metabolic activity, and the response was not dependent on cell density (5000 or 10,000 cells) (Figures 2a and S5a).

**Figure 3:**
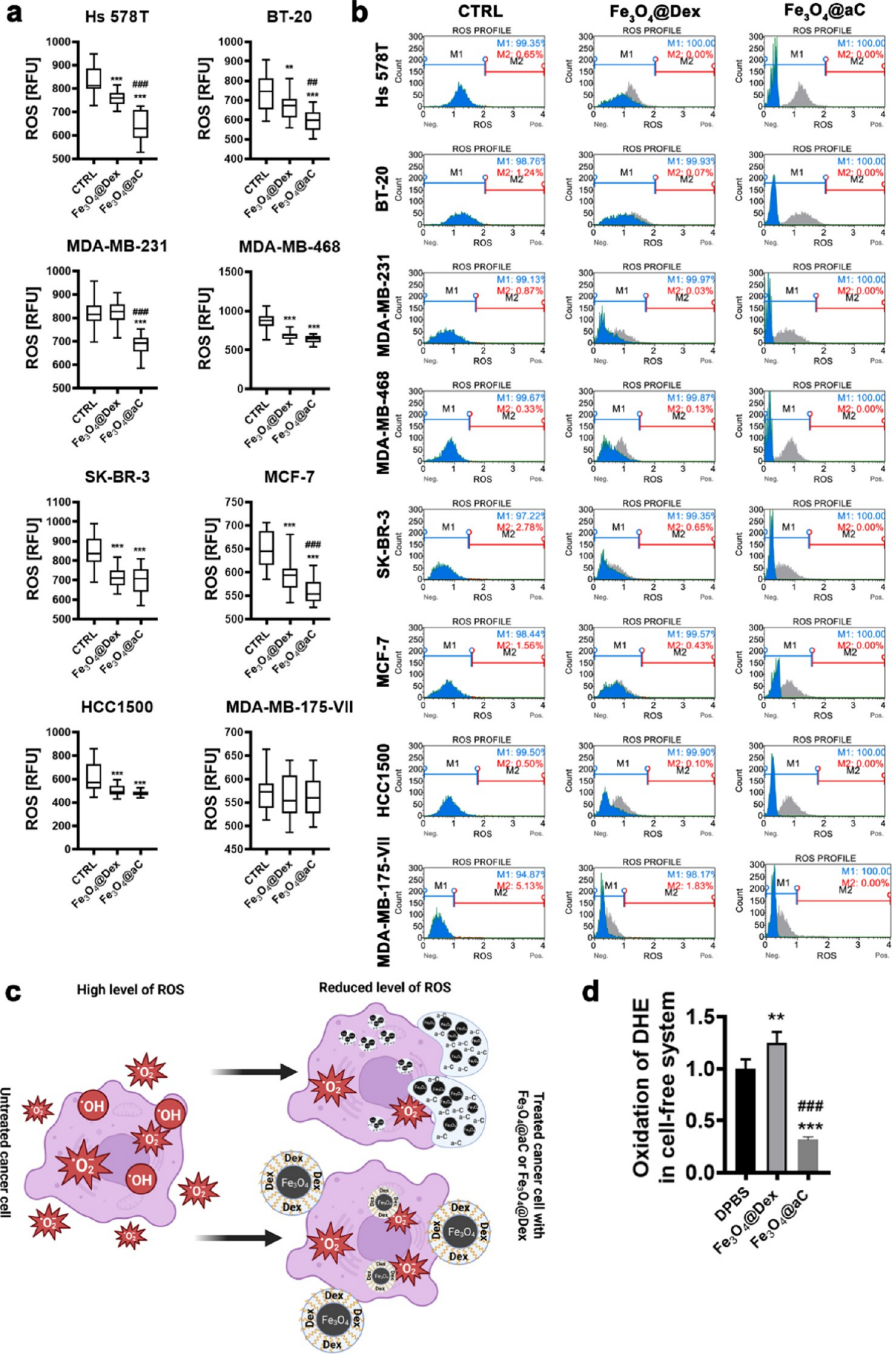
Redox imbalance promoted by encapsulated Fe_3_O_4_ NPs in breast cancer cells (a–c) and redox activity of encapsulated Fe_3_O_4_ NPs in a cell-free *in vitro* system (d). Cells were treated with 100 μg/mL NPs for 4 h, and total ROS levels (a) and total superoxide levels were analyzed using dedicated fluorogenic probes and imaging and flow cytometry, respectively. (a) Total ROS levels are presented in relative fluorescence units (RFU). Box and whisker plots are shown, *n* = 3, ***p < 0.001, and **p < 0.01 com*p*ared to untreated control (ANOVA and Dunnett’s a posteriori test); ^###^*p* < 0.001 and ^##^*p* < 0.01 compared to Fe_3_O_4_@Dex treatment (ANOVA and Tukey’s a posteriori test). (b) Representative histograms are shown. Superoxide levels are denoted as ROS. Blue histogram (M1), superoxide-negative population; red histogram (M2), superoxide-positive population; and gray histogram, ROS profile at control untreated conditions. (c) Graph illustrating reductive stress induced by encapsulated Fe_3_O_4_ NPs in breast cancer cells. Created with BioRender.com. (d) Dihydroethidium-based fluorescence in DPBS in the presence and the absence of encapsulated Fe_3_O_4_ NPs. Data were normalized to control (relative fluorescence units, RFU of dihydroethidium in DPBS). Bars indicate SD, *n* = 3, ***p < 0.001, and **p < 0.01 compared to dihydroethidium in DPBS (ANOVA and Dunnett’s a posteriori test); ^###^*p* < 0.001 compared to Fe_3_O_4_@Dex action (ANOVA and Tukey’s a posteriori test). CTRL, untreated control; DHE, dihydroethidium; DPBS, Dulbecco’s phosphate-buffered saline; Fe_3_O_4_@Dex, dextran-based coated iron oxide nanoparticles; and Fe_3_O_4_@aC, glucosamine-based amorphous carbon-coated iron oxide nanoparticles.

In contrast, some minor to moderate but statistically significant decrease in metabolic activity was noted when 100 μg/mL encapsulated Fe_3_O_4_ NPs were used (Hs 578T, BT-20, and MDA-MB-468 cells, Figure 2a). Surprisingly, metabolic activity was stimulated when HCC1500 cells were treated with 100 μg/mL Fe_3_O_4_@Dex and Fe_3_O_4_@aC (Figure 2a). As prolonged treatment (up to 24 h) did not affect substantially metabolic activity compared to 4 h treatment (Figure S5b), we decided to select the incubation time of 4 h and a 100 μg/mL concentration of encapsulated Fe_3_O_4_ NPs for further analysis. Normal human cells, namely, nontumorigenic epithelial cells originated from the mammary gland (MCF 10F cells) and skin fibroblasts (BJ cells), were also not more susceptible to Fe_3_O_4_@Dex and Fe_3_O_4_@aC treatment compared to breast cancer cells as judged by MTT results (Figure S6a). We assessed then the uptake of NPs using acridine orange staining of lysosomes upon stimulation with NPs (Figure 2b). We found that the cells differently responded to the treatment with NPs in the context of the affected lysosomal activity, namely, Fe_3_O_4_@aC-treated Hs 578T, MDA-MB-231, MDA-MB-468, SK-BR-3, MCF-7, and HC1500 cells were characterized by increased lysosomal activity, whereas the stimulation with NPs did not affect the activity of lysosomes in BT-20 and MDA-MB-175-VII cells (Figure 2b). Perhaps the analysis of lysosomal activity did not reflect the uptake of NPs in all breast cancer cell lines studied (Figure 2b). We next analyzed the intracellular production of total ROS upon stimulation with encapsulated Fe_3_O_4_ NPs (Figure 3a) as glucosamine-based carbon coating may have potential reductive activity in biological systems (*e.g.*, free radical scavenging ability) due to the presence of C=C, C–OH, and C=O groups.^48−51^

Indeed, except for the MDA-MB-175-VII cell line, in Fe_3_O_4_@aC-treated cells, the levels of ROS were significantly decreased compared to untreated conditions (Figure 3a). A mild to moderate reductive activity of Fe_3_O_4_@Dex was also observed in selected cell lines, but the effect of Fe_3_O_4_@aC was much more pronounced (Figure 3a). The levels of superoxide were also assayed (Figure 3b). Fe_3_O_4_@aC treatment dramatically affected the cell-based histograms (blue histograms, Figure 3b) compared to control conditions. Some very slight effects were also noted upon the stimulation with Fe_3_O_4_@Dex (Figure 3b,c). The reductive activity of Fe_3_O_4_@aC was also confirmed in a cell-free system *in vitro*, namely, upon incubation with a superoxide-specific fluorogenic probe (dihydroethidium) in a dedicated buffer (Figure 3d). Similar results were not obtained in the case of Fe_3_O_4_@Dex, as the presence of dextran slightly promoted the oxidation state of DHE in the cell-free system (Figure 3d).

As presented, the Fe_3_O_4_@aC NPs show unusual reductive properties, manifesting in the ROS reduction instead of increasing their concentration. The literature data indicate that using Fe_3_O_4_ NPs mainly results in increased ROS production, which is related to the leaching of iron ions from their surface.^12^ Accordingly, it is observed herein that the opposite effect should be related to the protection of the Fe_3_O_4_ surface by a hydrochar-like shell. The Fe ions on the magnetite surface are inactive, the leaching process cannot appear, and the carbon-based shell rich in oxygen-containing groups and acts more likely to scavenge the ROS than initiate their formation. To confirm this finding, the scavenging effect of Fe_3_O_4_@aC was tested. The scavengers are mainly used in catalysis to determine the production of the different radicals such as HOO^•^, HO^•^, and O_2_^•–^.^52^ Accordingly, this study applied a similar procedure, where nanoparticles were tested as scavengers of these radicals. More details about the applied method can be found in the Supporting Information. The tests were performed for model organic dye—Rhodamine B (RhB). According to the FTIR and XPS spectra analysis and adsorption tests (see the Supporting Information), the surface of the Fe_3_O_4_@aC hydroxyl, epoxy, and reactive free carbon sites should be responsible for their scavenging properties.

To confirm that, a small amount of nanoparticles (to avoid the adsorption of whole RhB) was added into the UV/H_2_O_2_ system, and the RhB degradation rate was measured and compared with the system without the addition of nanoparticles (Figure S7). Generally, when magnetite is added to the solution, the degradation rate should increase, which is related to generating, among others, HO^•^ radicals in the Fenton reaction.^52^ However, in the analyzed system, the degradation rate decreases from 20.18 to 13.15%. Accordingly, the sample does not increase the HO^•^ generation process but acts like a scavenger and reacts with the radicals. Normally, the addition of Fe-ion sources (such as magnetite) should increase the degradation rate by privileging the Fenton process; however, an opposite scenario (compatible with the findings from the biological studies) was observed. In the first stage, RhB is adsorbed on the Fe_3_O_4_@aC surface. Afterward, the highly reactive HO^•^ reacts, among others, with the free carbon sites, which results in the desorption process of RhB molecules. This reaction is followed by the further reaction of these radicals with an amorphous carbon shell and then with the RhB molecules. This reaction, schematically described by reactions 1–5, forms the hydroxyl functional groups (electrophilic addition) and oxidizes the presented carbon shell functional groups. Reaction 1 is the primary reaction, which is responsible for the scavenging process according to recent studies,^49,51^ while the oxidation of the functional groups can also appear^42^ and be confirmed in the simulated environment rich in the ROS generated by the UV/H_2_O_2_ reaction (Figure S8a). Moreover, a mild to moderate reductive activity of Fe_3_O_4_@Dex observed in selected cell lines was also confirmed, and according to the FTIR study, the sample before and after the reaction in a ROS-rich environment is related to the oxidation of the dextran shell and strictly related to the Fenton process of the degradation of polymeric shell (Figure S8b).
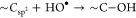
1
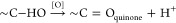
2
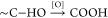
3
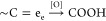
4
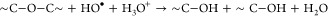
5where ∼C represents the carbon atoms in the amorphous carbon shell in Fe_3_O_4_@aC.

**Figure 4:**
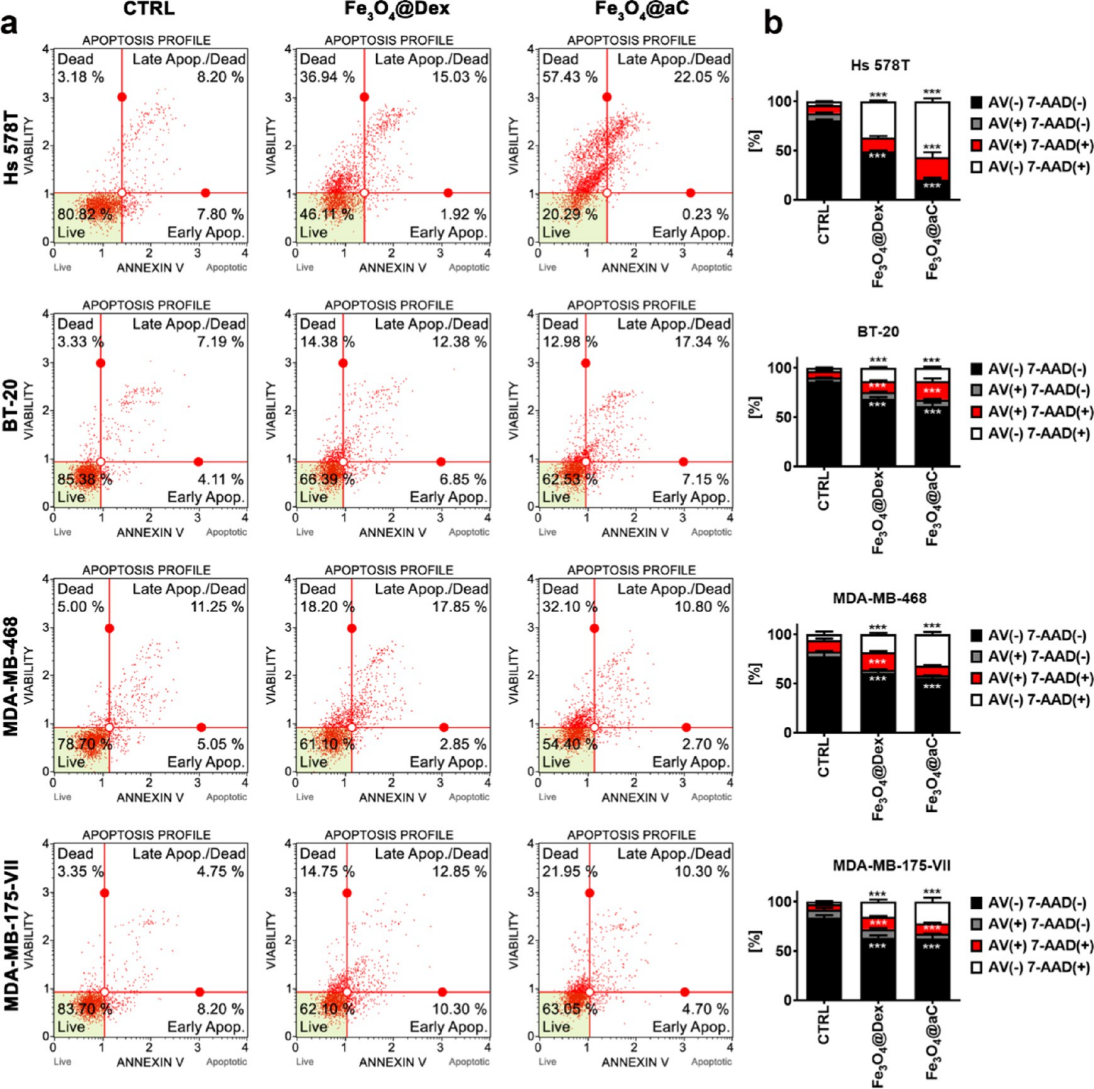
Apoptosis and necrosis induced by encapsulated Fe_3_O_4_ NPs in breast cancer cells. Cells were treated with 100 μg/mL NPs for 4 h, and apoptotic and necrotic cell death was revealed using Annexin V (phosphatidylserine externalization, an apoptotic marker) and 7-AAD (rupture of the plasma membrane, a necrotic marker) dual staining and flow cytometry. (a) Representative dot plots are shown. (b) Bars indicate SD, *n* = 3, and ***p < 0.001 compared to untreated control (ANOVA and Dunnett’s a posteriori test). Four subpopulations are shown, namely, live cells (Annexin V (AV)-negative, 7-AAD-negative), early apoptotic cells (Annexin V (AV)-positive, 7-AAD-negative), late apoptotic cells (Annexin V (AV)-positive, 7-AAD-positive), and necrotic cells (Annexin V (AV)-negative, 7-AAD-positive). CTRL, untreated control; Fe_3_O_4_@Dex, dextran-based coated iron oxide nanoparticles; and Fe_3_O_4_@aC, glucosamine-based amorphous carbon-coated iron oxide nanoparticles.

For further analysis, four cell lines were selected, namely, Hs 578T, BT-20, MDA-MB-468, and MDA-MB-175-VII cells on the basis of diverse response to Fe_3_O_4_@aC treatment in terms of changes in lysosomal activity (Figure 2b) and ROS production (Figure 3a). In general, Fe_3_O_4_@aC was more cytotoxic than Fe_3_O_4_@Dex in the context of Fe_3_O_4_@aC-mediated apoptotic and necrotic cell death (Figure 4). Fe_3_O_4_@aC was also more cytotoxic to normal cells than Fe_3_O_4_@Dex (Figure S6b). However, normal cells were less susceptible to Fe_3_O_4_@Dex treatment compared to breast cancer cells (Figure S6b). Hs 578T cells with the most pronounced Fe_3_O_4_@aC-associated reductive stress (Figure 3a) were also the most sensitive to Fe_3_O_4_@aC-induced cell death (Figure 4).

Perhaps in three breast cancer cell lines (Hs 578T, BT-20, and MDA-MB-468 cells), Fe_3_O_4_@aC-mediated cytotoxicity is associated with reductive stress (Figures 3a and 4). In contrast, in MDA-MB-175-VII cells with unaffected levels of ROS upon the stimulation with NPs, a similar response to both treatments with Fe_3_O_4_@Dex and Fe_3_O_4_@aC was observed (Figures 3a and 4). Perhaps, in these cells, Fe_3_O_4_@aC-induced cell death is executed by other mechanisms.

### Etoposide-Induced Senescent Breast Cancer Cells are Sensitive to Fe_3_O_4_@aC-Mediated Cell Death

**Figure 5:**
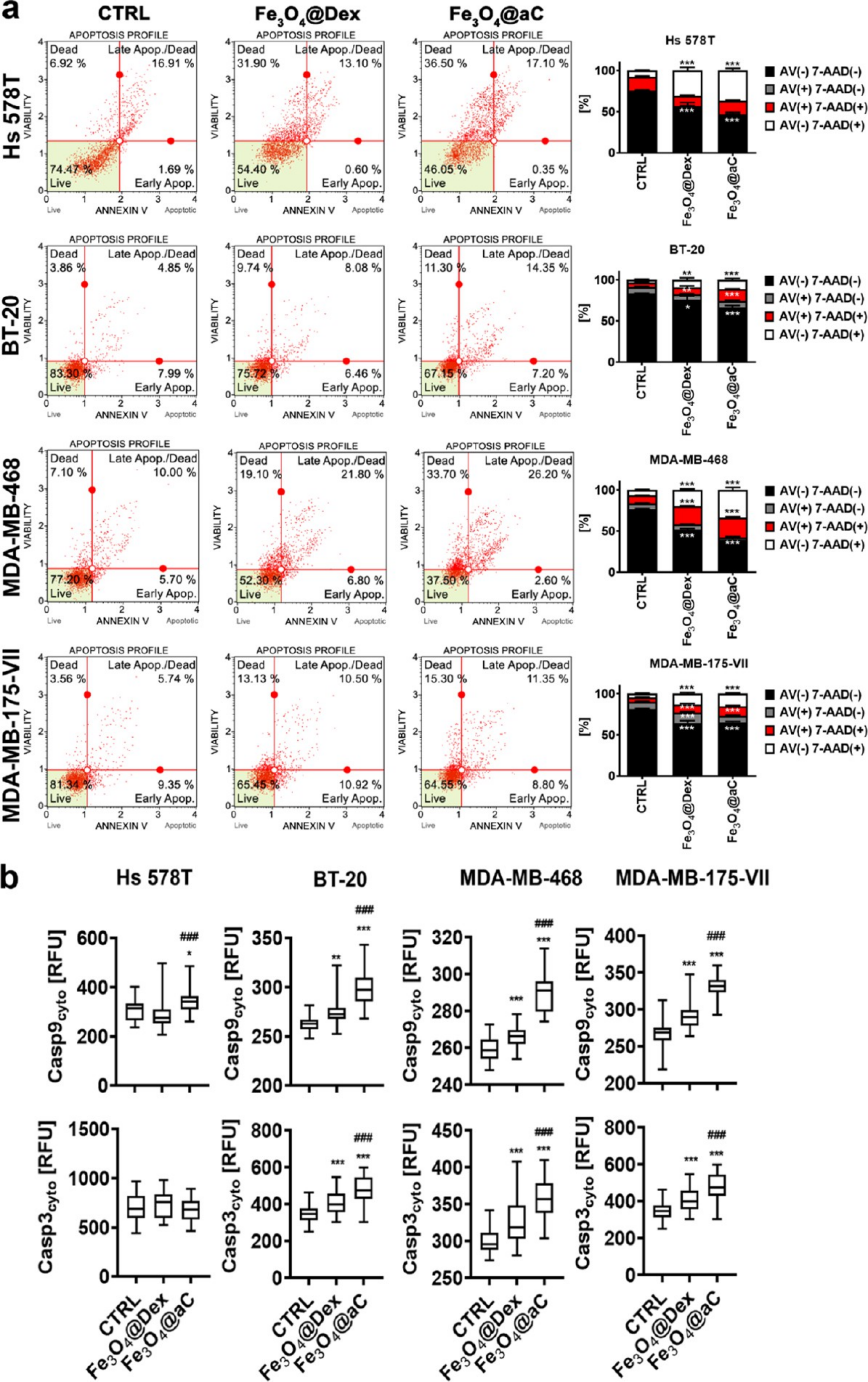
Apoptosis and necrosis induced by encapsulated Fe_3_O_4_ NPs in drug-induced senescent breast cancer cells. The senescence program was activated using etoposide treatment. Senescent breast cancer cells were treated with 100 μg/mL NPs for 4 h, and apoptotic and necrotic cell death was revealed using Annexin V (phosphatidylserine externalization, an apoptotic marker) and 7-AAD (rupture of plasma membrane, a necrotic marker) dual staining and flow cytometry (a). Representative dot plots are shown. (b) Bars indicate SD, *n* = 3, ***p < 0.001, **p < 0.01, and *p < 0.05 compared to untreated control (ANOVA and Dunnett’s a posteriori test). Four subpopulations are shown, namely, live cells (Annexin V (AV)-negative, 7-AAD-negative), early apoptotic cells (Annexin V (AV)-positive, 7-AAD-negative), late apoptotic cells (Annexin V (AV)-positive, 7-AAD-positive), and necrotic cells (Annexin V (AV)-negative, 7-AAD-positive). (b) Apoptosis was also studied using the analysis of the levels of caspase 9 and caspase 3. The levels of caspase 9 and caspase 3 were assayed using immunostaining and imaging cytometry. The levels of caspase 9 and caspase 3 are presented as relative fluorescence units (RFU). Box and whisker plots are shown, *n* = 3, ***p < 0.001, **p < 0.01, and *p < 0.05 compared to untreated control (ANOVA and Dunnett’s a posteriori test); ^###^*p* < 0.001 compared to Fe_3_O_4_@Dex treatment (ANOVA and Tukey’s a posteriori test). CTRL, untreated control; Fe_3_O_4_@Dex, dextran-based coated iron oxide nanoparticles; and Fe_3_O_4_@aC, glucosamine-based amorphous carbon-coated iron oxide nanoparticles.

Cellular senescence is considered to be a side effect of chemotherapy resulting in the induction of secondary senescence in normal and cancer cells, promotion of cancer cell proliferation, migration, and invasiveness, and immune responses in a tumor microenvironment and drug resistance leading to limited success of anticancer drug treatment(s).^3,53^ Thus, we decided then to also analyze the effects of Fe_3_O_4_@Dex and Fe_3_O_4_@aC in drug-induced senescent breast cancer cells. A cellular model of chemotherapy-induced senescence was used, namely, the activation of the senescence program upon stimulation with etoposide, an anticancer drug.^33^ Cellular senescence did not affect the response to Fe_3_O_4_@Dex and Fe_3_O_4_@aC in BT-20 and MDA-MB-175-VII cells (Figure 5a). In contrast, senescent MDA-MB-468 cells were more sensitive to Fe_3_O_4_@aC treatment than nonsenescent MDA-MB-468 cells, and senescent Hs 578T cells were less sensitive to Fe_3_O_4_@aC treatment than nonsenescent Hs 578T cells (Figures 4 and 5a). Fe_3_O_4_@aC-induced apoptosis in senescent breast cancer cells was then more comprehensively evaluated (Figure 5b). The levels of caspase 9 (an initiator caspase involved in the induction of the mitochondrial pathway of apoptosis) and caspase 3 (a major executioner caspase) were assayed (Figure 5b). The levels of caspase 9 were elevated in Fe_3_O_4_@aC-treated senescent breast cancer cells compared to untreated senescent cells but also increased compared to Fe_3_O_4_@Dex-treated senescent cells (Figure 5b).

**Figure 6:**
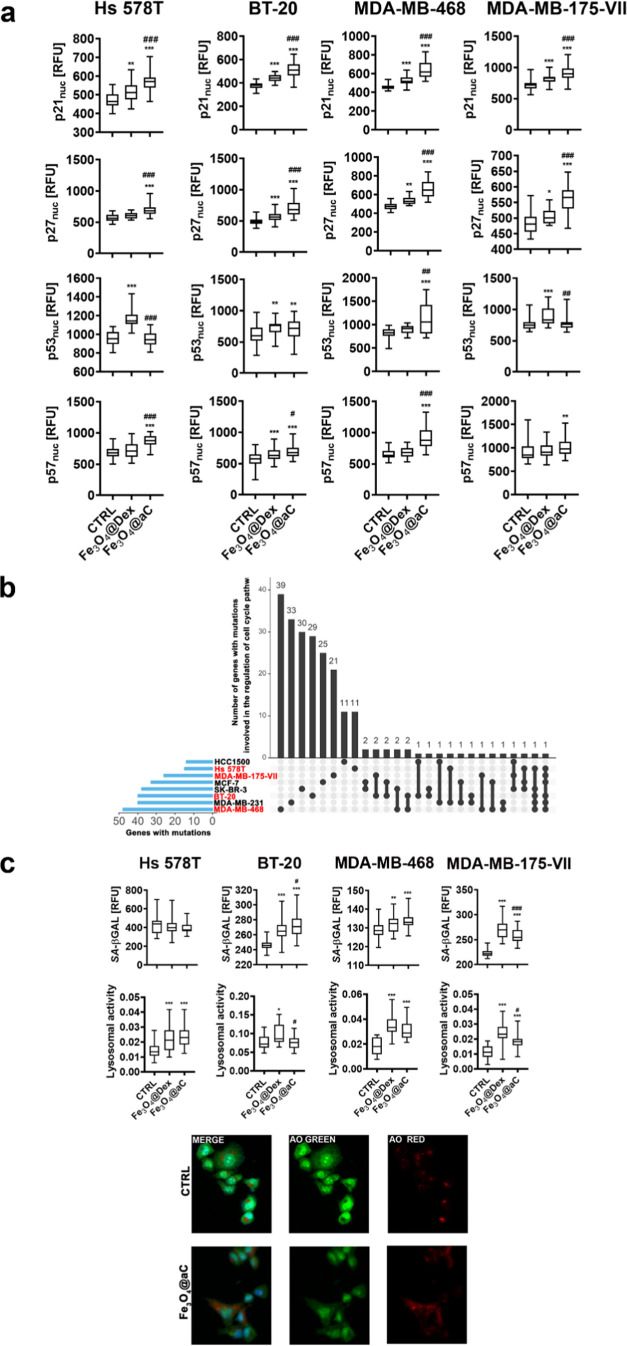
Effects of encapsulated Fe_3_O_4_ NPs on the levels of cell cycle inhibitors (a) and senescence-associated-β-galactosidase (SA-βGAL) and lysosomal activity (c) in drug-induced senescent breast cancer cells. The number of gene mutations involved in the regulation of cell cycle pathways in studied cell lines (in red) is also shown (b). The senescence program was activated using etoposide treatment. Senescent breast cancer cells were treated with 100 μg/mL NPs for 4 h. (a) Nuclear pools of p21, p27, p53, and p57 were analyzed using immunostaining and imaging cytometry. The levels of p21, p27, p53, and p57 are presented as relative fluorescence units (RFU). Box and whisker plots are shown, *n* = 3, ***p < 0.001, **p < 0.01, and *p < 0.05 compared to untreated control (ANOVA and Dunnett’s a posteriori test); ^###^*p* < 0.001, ^##^*p* < 0.01, and ^#^*p* < 0.05 compared to Fe_3_O_4_@Dex treatment (ANOVA and Tukey’s a posteriori test). (b) Gene mutation raw data were downloaded from the DepMap portal (https://depmap.org/portal/). Set intersections in a matrix layout were visualized using the UpSet plot. Total, shared, and unique gene mutations in genes involved in the regulation of the cell cycle across eight breast cancer cell lines are presented. Blue bars in the *y*-axis denote the total number of gene mutations in each cell line. Black bars in the *x*-axis denote the number of mutations shared across cell lines connected by the black dots in the body of the plot. (c) SA-βGAL activity was analyzed using a dedicated staining kit and imaging cytometry. SA-βGAL activity is presented in relative fluorescence units (RFU). The uptake of NPs was assessed as changes in lysosomal activity. The lysosomal activity was revealed using acridine orange staining and imaging cytometry. Representative microphotographs are presented (green, red and merged channels are included). The lysosomal activity was calculated according to the formula: the number of lysosomes per cells/red channel fluorescence × lysosome area. Box and whisker plots are shown, *n* = 3, ***p < 0.001, **p < 0.01, and *p < 0.05 compared to untreated control (ANOVA and Dunnett’s a posteriori test); ^###^*p* < 0.001 and ^#^*p* < 0.05 compared to Fe_3_O_4_@Dex treatment (ANOVA and Tukey’s a posteriori test). CTRL, untreated control; Fe_3_O_4_@Dex, dextran-based coated iron oxide nanoparticles; Fe_3_O_4_@aC, glucosamine-based amorphous carbon-coated iron oxide nanoparticles; and AO, acridine orange staining.

Except for senescent Hs 578T cells, similar effects were observed in Fe_3_O_4_@aC-treated senescent breast cancer cells in the case of the levels of caspase 3 (Figure 5b). As cellular senescence is characterized by increased levels of cell cycle inhibitors,^53^ selected cell cycle regulators were also studied upon stimulation with Fe_3_O_4_@Dex and Fe_3_O_4_@aC in senescent breast cancer cells (Figure 6a). The nuclear pools of p21, p27, and p57 were elevated in Fe_3_O_4_@aC-stimulated senescent breast cancer cells compared to both untreated and Fe_3_O_4_@Dex-treated senescent cells (Figure 6a). Perhaps in our experimental settings, an increase in the levels of cell cycle inhibitors was associated with Fe_3_O_4_@aC-mediated cell death response in senescent cells (Figure 6a). In Fe_3_O_4_@aC-treated senescent MDA-MB-468 cells, also the levels of nuclear p53 were augmented compared to control conditions and Fe_3_O_4_@Dex treatment (Figure 6a). The levels of gene mutations in genes involved in the regulation of the cell cycle pathway did not correlate with cell cycle inhibitor-based response and Fe_3_O_4_@aC-mediated cytotoxicity as MDA-MB-468 cells with the highest levels of cell cycle-related gene mutations and Hs 578T cells with the lowest levels of cell cycle-related gene mutations (Figure 6b, in red; a detailed list of mutated genes can be found in Table S1, Supporting Information) were the most susceptible to Fe_3_O_4_@aC treatment upon activation of the senescence program (Figure 5a).

Another marker of cellular senescence was also analyzed upon the stimulation with encapsulated NPs, namely, senescence-associated β-galactosidase (SA-βGAL) activity^54^ (Figure 6c). Except for Hs 578T cells, the levels of SA-βGAL-positive cells were increased upon treatment with Fe_3_O_4_@Dex and Fe_3_O_4_@aC (Figure 6c). However, one should remember that, in contrast to the majority of normal cells, SA-βGAL activity might not be always associated with cellular senescence in cancer cells as in nonsenescent cancer cells, this marker might be also elevated.^55^ We also correlated SA-βGAL activity results with lysosomal activity using acridine orange staining^31^ (Figure 6c). The lysosomal activity was elevated in Fe_3_O_4_@Dex and Fe_3_O_4_@aC-treated senescent Hs 578T, MDA-MB-468, and MDA-MB-175-VII cells; thus, in senescent MDA-MB-468 and MDA-MB-175-VII cells, SA-βGAL activity can be correlated with lysosomal activity upon the stimulation with encapsulated NPs (Figure 6c). This is in agreement with previous findings documenting that SA-β-galactosidase is lysosomal β-galactosidase.^56^

### Fe_3_O_4_@aC Promotes Reductive Stress and Cytotoxic Autophagy in Drug-Induced Senescent Breast Cancer Cells

**Figure 7:**
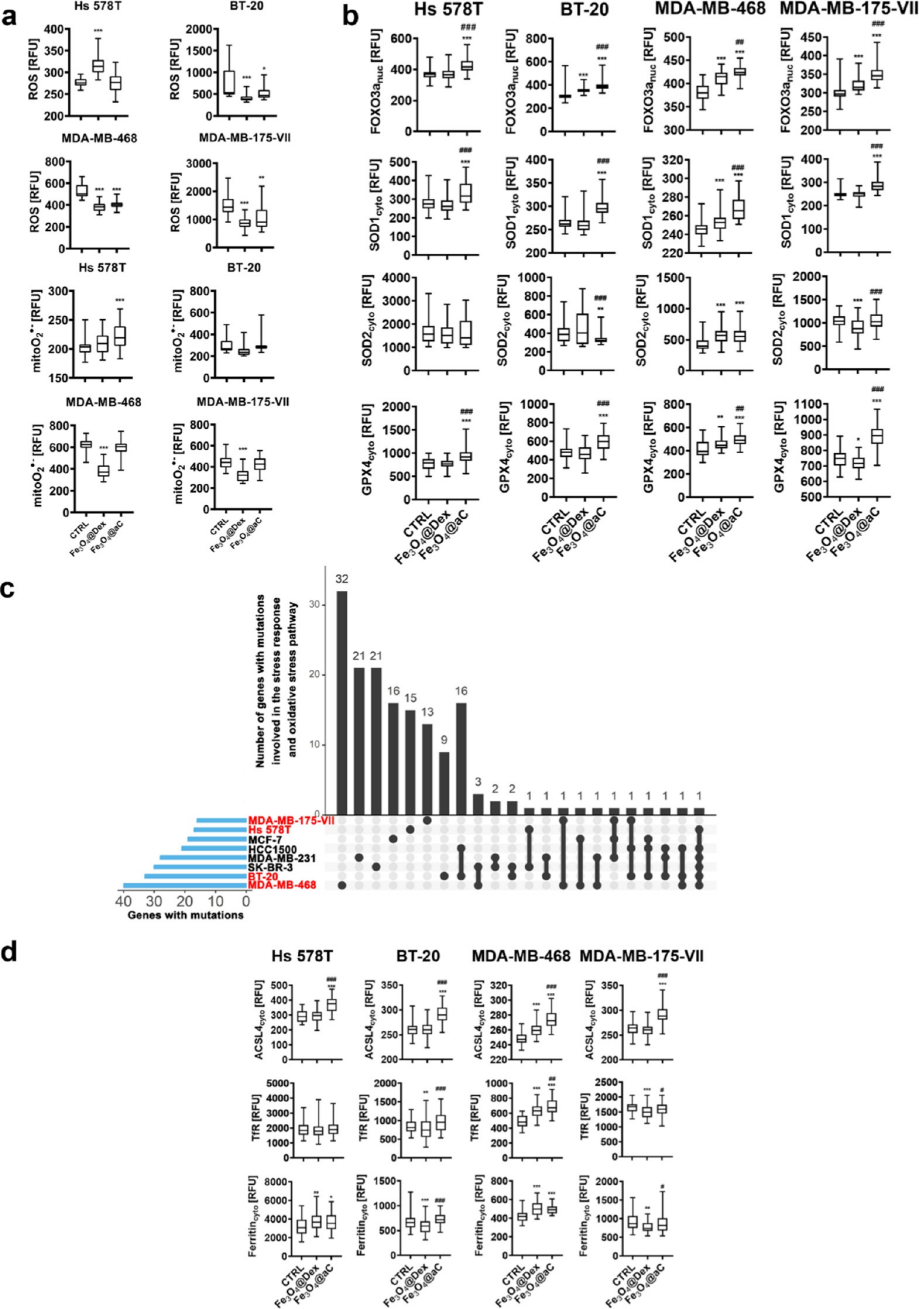
Induction of reductive stress (a, b) and changes in iron-mediated adaptive responses (d) in drug-induced senescent breast cancer cells treated with encapsulated Fe_3_O_4_ NPs. The number of gene mutations involved in the regulation of stress responses in studied cell lines (in red) is also shown (c). The senescence program was activated using etoposide treatment. Senescent breast cancer cells were treated with 100 μg/mL NPs for 4 h. (a) Levels of total ROS and mitochondrial superoxide were analyzed in live cells using dedicated fluorogenic probes and imaging cytometry. (b) Levels of FOXO3a, SOD1, SOD2, and GPX4 were analyzed in fixed cells using immunostaining and imaging cytometry. The levels of total ROS, mitochondrial superoxide (a), and antioxidant proteins, namely, nuclear FOXO3a and cytoplasmic SOD1, SOD2, and GPX4 (b), are presented in relative fluorescence units (RFU). (c) Gene mutation raw data were downloaded from the DepMap portal (https://depmap.org/portal/). Set intersections in a matrix layout were visualized using the UpSet plot. Total, shared, and unique gene mutations in genes involved in the regulation of stress responses across eight breast cancer cell lines are presented. Blue bars in the *y*-axis denote the total number of gene mutations in each cell line. Black bars in the *x*-axis denote the number of mutations shared across cell lines connected by the black dots in the body of the plot. (d) Levels of ACSL4, TfR, and ferritin were analyzed in fixed cells using immunostaining and imaging cytometry. The levels of ACSL4, TfR, and ferritin are presented in relative fluorescence units (RFU). (a, b, d) Box and whisker plots are shown, *n* = 3, ***p < 0.001, **p < 0.01, and *p < 0.05 compared to untreated control (ANOVA and Dunnett’s a posteriori test); ^###^*p* < 0.001, ^##^*p* < 0.01, and ^#^*p* < 0.05 compared to Fe_3_O_4_@Dex treatment (ANOVA and Tukey’s a posteriori test). CTRL, untreated control; Fe_3_O_4_@Dex, dextran-based coated iron oxide nanoparticles; and Fe_3_O_4_@aC, glucosamine-based amorphous carbon-coated iron oxide nanoparticles.

Fe_3_O_4_@aC-induced reductive stress was also studied in etoposide-induced senescent breast cancer cells (Figure 7). Indeed, except for senescent Hs 578T cells, Fe_3_O_4_@aC caused a decrease in the production of total ROS (Figure 7a). However, this diminution was not accompanied by reduced levels of mitochondrial superoxide (Figure 7a). We analyzed then antioxidant-based responses in more detail (Figure 7b). The levels of the nuclear pool of FOXO3a, a transcription factor involved in the regulation of antioxidant gene expression,^57^ and antioxidant enzymes, namely, superoxide dismutase 1 and 2 (SOD1 and SOD2) and glutathione peroxidase 4 (GPX4, phospholipid hydroperoxidase), were studied (Figure 7b). Fe_3_O_4_@aC promoted an increase in the levels of FOXO3a, SOD1, and GPX4 compared to both control conditions and Fe_3_O_4_@Dex treatment (Figure 7b). The gene mutation status in genes involved in the regulation of cellular stress responses, namely, oxidative stress response (Figure 7c; a detailed list of mutated genes can be found in Table S2, Supporting Information), did not modulate redox stress parameters as breast cancer cells with the highest and lowest number of stress response-related gene mutations responded similarly to Fe_3_O_4_@aC treatment (Figure 7b). Redox imbalance in cancer cells was mainly studied in the context of oxidative stress, and little is known if also reductive stress, an increase in reducing equivalents such as NADH, may have also therapeutic effects.^17^ It is widely accepted that cancer cells can cope with oxidative stress by the activation of the nuclear factor erythroid 2-related factor 2 (Nrf2)/Kelch-like ECH-associated protein 1 (Keap1) pathway that promotes the antioxidant response, ROS detoxification, and tumorigenesis.^58,59^ However, hyperactivation of Nrf2 may also result in sustained activation of antioxidant genes and reductive stress.^17^

For example, Nrf2 overactivation in breast cancer stem-like cells stimulated reductive stress and FOXO3a-mediated self-renewal activity and cell growth.^60^ In our experimental conditions, Fe_3_O_4_@aC-induced FOXO3a activation was accompanied by apoptotic cell death in breast cancer cells that suggests that FOXO3a-associated reductive stress and related cytotoxicity may depend on cellular context. More recently, Nrf2 activation-based NADH-reductive stress was also reported to promote metabolic vulnerability and limit cell proliferation in a subset of nonsmall cell lung cancer (NSCLC) cell lines.^18^ However, more studies are needed to document therapeutic potential and related mechanisms of reductive stress induction in cancer cells.

**Figure 8:**
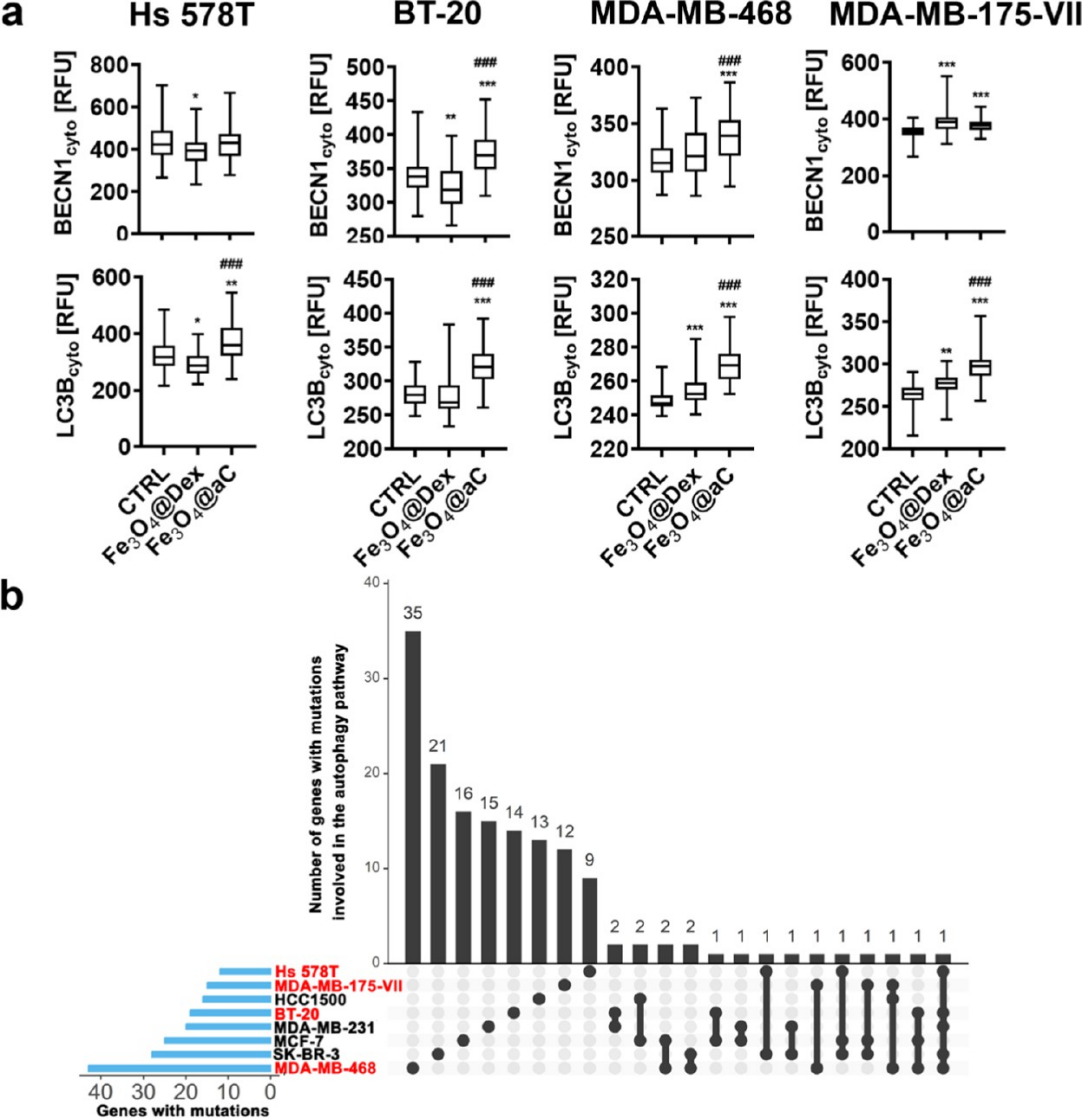
Autophagy induction in drug-induced senescent breast cancer cells treated with encapsulated Fe_3_O_4_ NPs (a). The number of gene mutations involved in the regulation of the autophagy pathway in studied cell lines (in red) is also shown (b). (a) Senescence program was activated using etoposide treatment. Senescent breast cancer cells were treated with 100 μg/mL NPs for 4 h. The cytoplasmic levels of BECN1 and LCB3 were analyzed in fixed cells using immunostaining and imaging cytometry. The levels of BECN1 and LCB3 are presented in relative fluorescence units (RFU). Box and whisker plots are shown, *n* = 3, ***p < 0.001, **p < 0.01, and *p < 0.05 compared to untreated control (ANOVA and Dunnett’s a posteriori test); ^###^*p* < 0.001 compared to Fe_3_O_4_@Dex treatment (ANOVA and Tukey’s a posteriori test). CTRL, untreated control; Fe_3_O_4_@Dex, dextran-based coated iron oxide nanoparticles; and Fe_3_O_4_@aC, glucosamine-based amorphous carbon-coated iron oxide nanoparticles. (b) Gene mutation raw data were downloaded from the DepMap portal (https://depmap.org/portal/). Set intersections in a matrix layout were visualized using the UpSet plot. Total, shared, and unique gene mutations in genes involved in the regulation of the autophagy pathway across eight breast cancer cell lines are presented. Blue bars in the *y*-axis denote the total number of gene mutations in each cell line. Black bars in the *x*-axis denote the number of mutations shared across cell lines connected by the black dots in the body of the plot.

GPX4 is also a marker of ferroptosis, an iron-dependent cell death,^61^ as decreased pools of GPX4 may promote ferroptotic cell death because of inadequate protection against iron-mediated lipid peroxidation. However, in our experimental conditions, Fe_3_O_4_@aC caused an increase in the levels of GPX4. Thus, perhaps Fe_3_O_4_@aC-treated cells were not vulnerable to ferroptotic cell death. This result inspired us to analyze more ferroptosis-related parameters and iron-mediated adaptive responses in drug-induced senescent breast cancer cells (Figure 7d). Surprisingly, Fe_3_O_4_@aC treatment resulted in the elevation of the levels of ACSL4 (acyl-CoA synthetase long-chain family member 4) in four senescent breast cancer cell lines (Figure 7d). Increased levels of ACSL4 may sensitize cells to ferroptosis,^62,63^ but in our experimental settings, this scenario is perhaps prevented by elevated levels of GPX4, a protector against iron-mediated lipid peroxidation (Figure 7d). The levels of transferrin receptor (TfR, iron (Fe^3+^) importer) and ferritin involved in intracellular iron storage were also analyzed upon Fe_3_O_4_@aC stimulation (Figure 7d). TfR was increased in Fe_3_O_4_@aC-treated senescent MDA-MB-468 cells, and ferritin levels were elevated in Fe_3_O_4_@aC-treated senescent Hs 578T and MDA-MB-468 cells (Figure 7d). Thus, senescent cells are indeed able to adapt to the presence of iron-based nanomaterial in a cell culture medium. Our data are in agreement with previously published results on iron-induced increases in the levels of TfR and ferritin in senescent mouse embryonic fibroblasts (MEFs).^64^ Furthermore, senescent MEFs, in control culture conditions without iron stimulation, were characterized by iron accumulation, impaired ferritinophagy (ferritin degradation), and inhibition of ferroptosis.^64^ However, forced activation of the ferritin degradation pathway by using autophagy inducer rapamycin did not resensitize senescent cells to ferroptosis.^64^ As we ruled out that Fe_3_O_4_@aC-mediated apoptosis and reductive stress may be accompanied by ferroptotic cell death in senescent breast cancer cells (Figures 5 and 7a), we asked then if cytotoxic autophagy may be also induced upon Fe_3_O_4_@aC stimulation in senescent breast cancer cells. Indeed, two markers of the autophagic pathway, namely, the levels of BECN1 and LC3B, were elevated in Fe_3_O_4_@aC-treated senescent cells (Figure 8a). Furthermore, the levels of LC3B were increased in Fe_3_O_4_@aC-treated senescent cells compared to Fe_3_O_4_@Dex-treated senescent cells (Figure 8a). The autophagic response was not affected by gene mutations in the autophagy pathway in studied cells as similar effects were observed in cells with the highest (MDA-MB-468 cell line) and the lowest (Hs 578T cell line) number of gene mutations in autophagy-related genes (Figure 8b; a detailed list of mutated genes can be found in Table S3, Supporting Information).

It has been documented that the induction of the autophagic pathway during chemotherapy may promote different cell responses such as cytotoxicity (cytotoxic autophagy), inhibition of cell proliferation (cytostatic autophagy), and inhibition of cell death and drug resistance (cytoprotective autophagy).^65^ As the induction of autophagy was accompanied by apoptotic cell death upon stimulation with Fe_3_O_4_@aC, one can conclude that cytotoxic autophagy was observed in our experimental conditions (Figures 5 and 8a).

### Fe_3_O_4_@aC Stimulates Proinflammatory Responses in Drug-Induced Senescent Breast Cancer Cells

**Figure 9:**
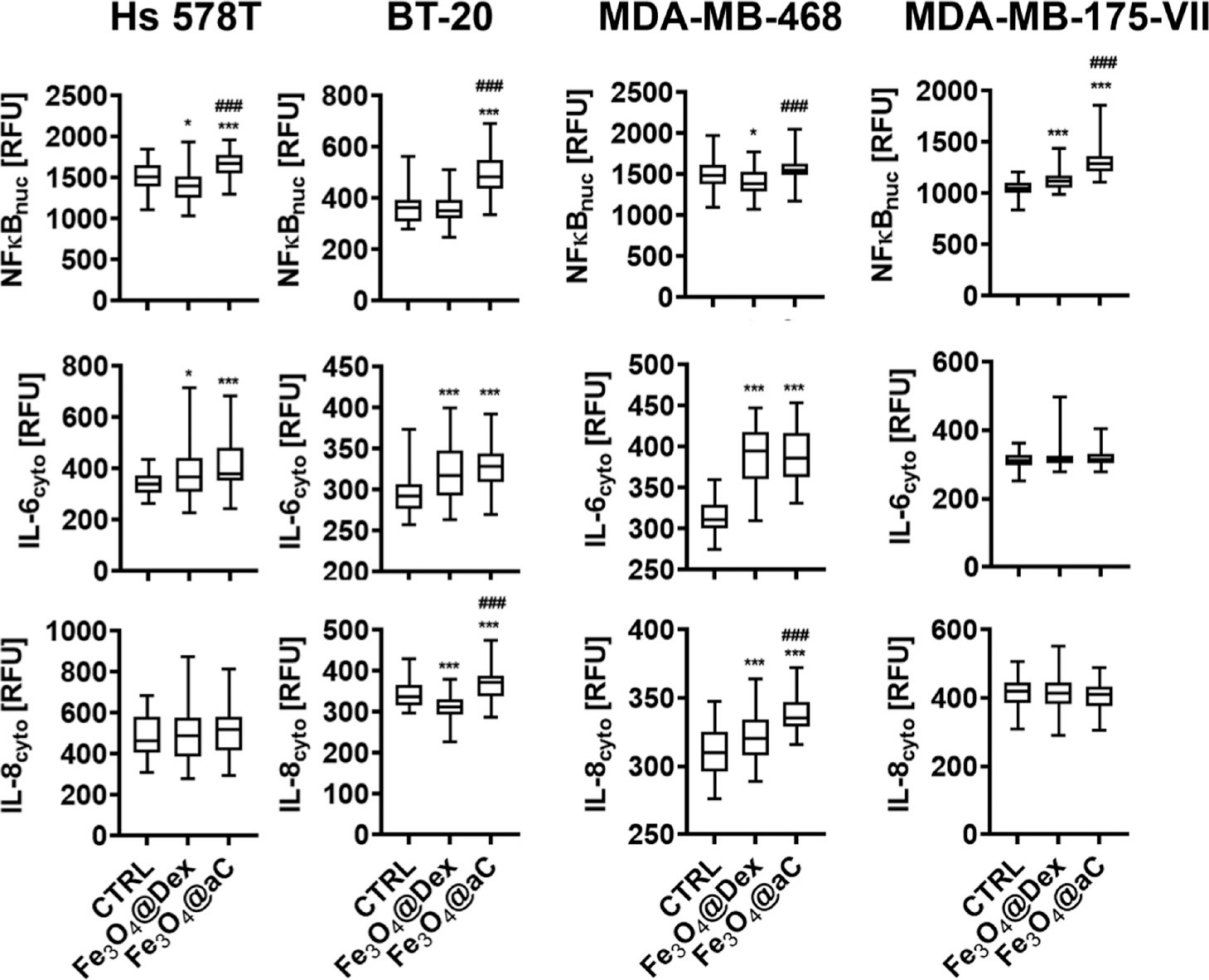
Proinflammatory response is stimulated in drug-induced senescent breast cancer cells treated with encapsulated Fe_3_O_4_ NPs. The senescence program was activated using etoposide treatment. Senescent breast cancer cells were treated with 100 μg/mL NPs for 4 h. The nuclear pools of NFκB and cytoplasmic levels of IL-6 and IL-8 were analyzed in fixed cells using immunostaining and imaging cytometry. The levels of NFκB, IL-6, and IL-8 are presented in relative fluorescence units (RFU). Box and whisker plots are shown, *n* = 3, ^***^p < 0.001, and *p < 0.05 com*p*ared to untreated control (ANOVA and Dunnett’s a posteriori test); ^###^*p* < 0.001 compared to Fe_3_O_4_@Dex treatment (ANOVA and Tukey’s a posteriori test). CTRL, untreated control; Fe_3_O_4_@Dex, dextran-based coated iron oxide nanoparticles; and Fe_3_O_4_@aC, glucosamine-based amorphous carbon-coated iron oxide nanoparticles.

As cellular senescence is accompanied by senescence-associated secretory phenotype (SASP), a secretion of proinflammatory factors that may promote inflammation in the tumor microenvironment stimulating cancer cell proliferation and secondary senescence,^4^ we also then studied the effects of encapsulated iron oxide NPs on the activation of a major regulator of immune responses, namely, nuclear levels of NFκB and the production of two proinflammatory cytokines, IL-6 and IL-8, in senescent breast cancer cells. NFκB activation was observed in four senescent breast cancer cell lines upon Fe_3_O_4_@aC stimulation (Figure 9). Furthermore, the nuclear levels of NFκB were increased in Fe_3_O_4_@aC-treated senescent cells compared to Fe_3_O_4_@Dex-treated senescent cells (Figure 9). Similar effects were obtained in terms of IL-8 production in Fe_3_O_4_@aC-treated BT-20 and MDA-MB-468 senescent cells (Figure 9). Except for the MDA-MB-175-VII cell line, treatment with encapsulated iron oxide NPs resulted in increased production of IL-6 in senescent breast cancer cells (Figure 9). Fe_3_O_4_@aC-mediated proinflammatory response (Figure 9) may also promote an increase in the levels of ferritin in senescent breast cancer cells (Figure 7d).^66^

### Fe_3_O_4_@aC Induces Nucleolar Stress and Changes in the Components of the Nuclear Lamina

**Figure 10:**
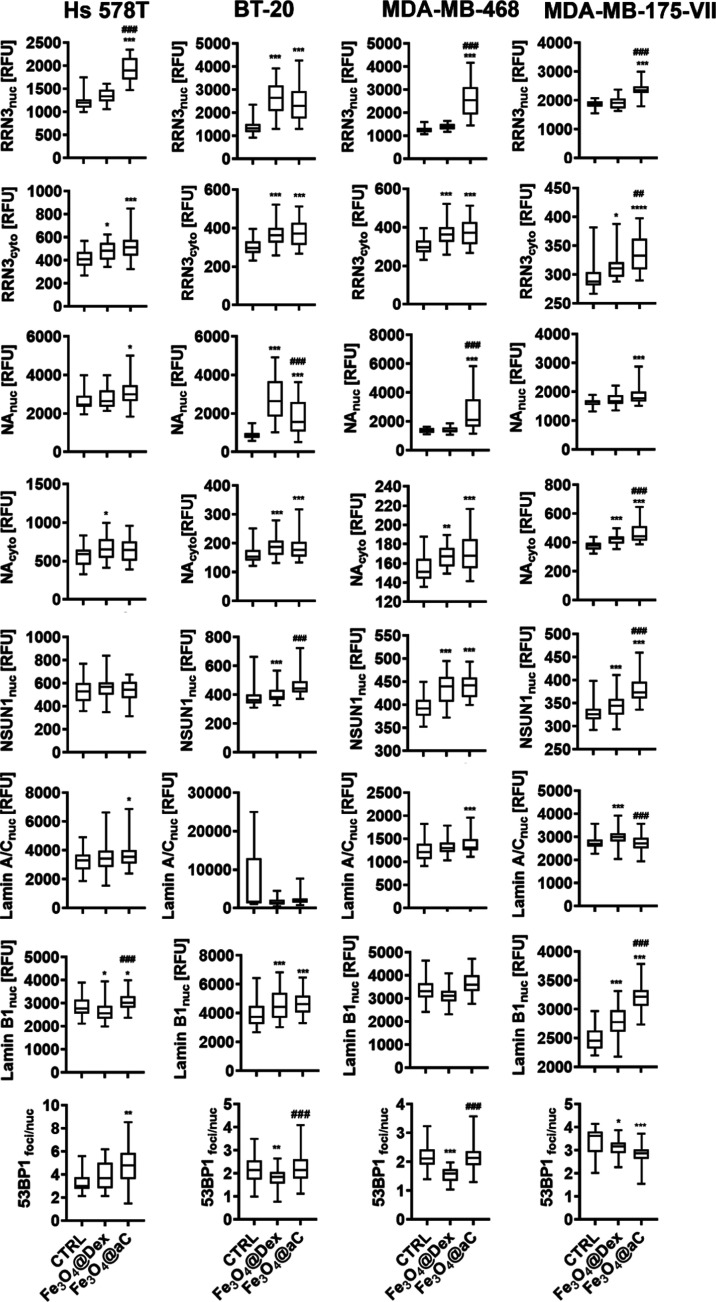
Induction of nucleolar stress and changes in the components of the nuclear lamina and DNA damage response (DDR) in drug-induced senescent breast cancer cells treated with encapsulated Fe_3_O_4_ NPs. The senescence program was activated using etoposide treatment. Senescent breast cancer cells were treated with 100 μg/mL NPs for 4 h. Nucleolar stress was studied as changes in the nuclear and cytoplasmic pools of RRN3, NA, and NOP2 (NSUN1). Changes in the components of the nuclear lamina were judged as the analysis of nuclear levels of lamin A/C and lamin B1. DDR was assayed as the formation of 53BP1 foci. The levels of RRN3, NA, NSUN1, lamin A/C, lamin B1, and 53BP1 foci were analyzed in fixed cells using immunostaining and imaging cytometry. The levels of RRN3, NA, NSUN1, lamin A/C, and lamin B1 are presented in relative fluorescence units (RFU). The formation of 53BP1 foci was calculated per nucleus. Box and whisker plots are shown, *n* = 3, ***p < 0.001, **p < 0.01, and *p < 0.05 compared to untreated control (ANOVA and Dunnett’s a posteriori test); ^###^*p* < 0.001 and ^##^*p* < 0.01 compared to Fe_3_O_4_@Dex treatment (ANOVA and Tukey’s a posteriori test). CTRL, untreated control; Fe_3_O_4_@Dex, dextran-based coated iron oxide nanoparticles; and Fe_3_O_4_@aC, glucosamine-based amorphous carbon-coated iron oxide nanoparticles.

As nucleolus is considered a stress sensor and regulator of adaptive responses during oxidative and ribotoxic stress,^67,68^ we decided then to analyze if Fe_3_O_4_@aC-mediated reductive stress may also promote nucleolus-based response in drug-induced senescent breast cancer cells (Figure 10). The nuclear fractions of nucleolus-related proteins (RRN3, NA, NSUN1/NOP2) were increased upon stimulation with Fe_3_O_4_@aC (Figure 10). Furthermore, cytoplasmic fractions of RRN3 and NA were also elevated in Fe_3_O_4_@aC-treated cells (Figure 10), which may suggest their relocation from the nucleolus to the cytoplasm.

Indeed, stress-induced relocalization of nucleolar proteins is a marker of nucleolar stress.^67,68^ For example, under stress conditions, the nuclear pools of Pol I-specific transcription factor RRN3/TIF-IA were diminished that inhibited RNA polymerase I (Pol I) transcription and rRNA synthesis and aberrant activity of RRN3 promoted nucleolar disruption, cell cycle arrest, and p53-mediated apoptosis.^68^ Perhaps Fe_3_O_4_@aC-induced nucleolar stress may also be associated with cytotoxic effects in drug-induced senescent breast cancer cells (this study).

As changes in the levels of nuclear lamins may also be associated with cellular senescence, genomic instability, and nucleolar stress,^69,70^ we analyzed then the levels of lamin A/C and B1 along with a marker of DNA damage response (DDR), the formation of 53BP1 foci in senescent breast cancer cells treated with encapsulated iron oxide NPs (Figure 10). Fe_3_O_4_@aC treatment resulted in an increase in the levels of lamin A/C and B1 in senescent breast cancer cells; however, only in Hs 578T cells, this effect was accompanied by increased production of 53BP1 foci (Figure 10) that may suggest that DNA damage and subsequent DDR may not account for Fe_3_O_4_@aC-mediated cytotoxicity. One can also conclude that both stress stimulus-mediated increase and decrease in the levels of nuclear lamins might affect the organization of the nuclear lamina and promote nucleolar stress in breast cancer cells (this study and ref (70)).

The present study has some limitations. We have documented Fe_3_O_4_@aC-mediated cytotoxicity using several cellular models of breast cancer *in vitro*. Thus, more studies are needed to evaluate the usefulness of Fe_3_O_4_@aC in *in vivo* systems, for example, using tumor xenograft animal models along with biocompatibility testing to exclude some unwanted side effects on normal healthy tissues. Furthermore, the mechanisms of reductive stress-mediated cytotoxicity after Fe_3_O_4_@aC treatment in cancer cells should be more comprehensively analyzed in terms of interconnections between Fe_3_O_4_@aC-mediated reductive stress, apoptosis, autophagy, and immune responses.

## Conclusions

In the present study, for the first time, we showed the possibility of synthesizing carbon-coated magnetite nanoparticles with numerous reactive oxygen-rich functional groups using the one-step polyol method and d-glucosamine sulfate potassium chloride as the carbon source. The proposed synthesis method allowed us to obtain superparamagnetic material (retentivity close to 0 emu/g and *H*_c_ equal to 0.27 Oe), in which ultrafine (7.85 ± 0.45 nm) nanoparticles are embedded into amorphous carbon with a structure similar to the hydrochar. For comparison, the superparamagnetic dextran 70,000 coated Fe_3_O_4_ NPs were synthesized using the same method. Superparamagnetic nanoparticles covered by an organic layer were characterized by a lower particle size equal to 6.33 ± 0.40 nm and higher saturation magnetization equal to 32.87 emu/g.

**Figure 11:**
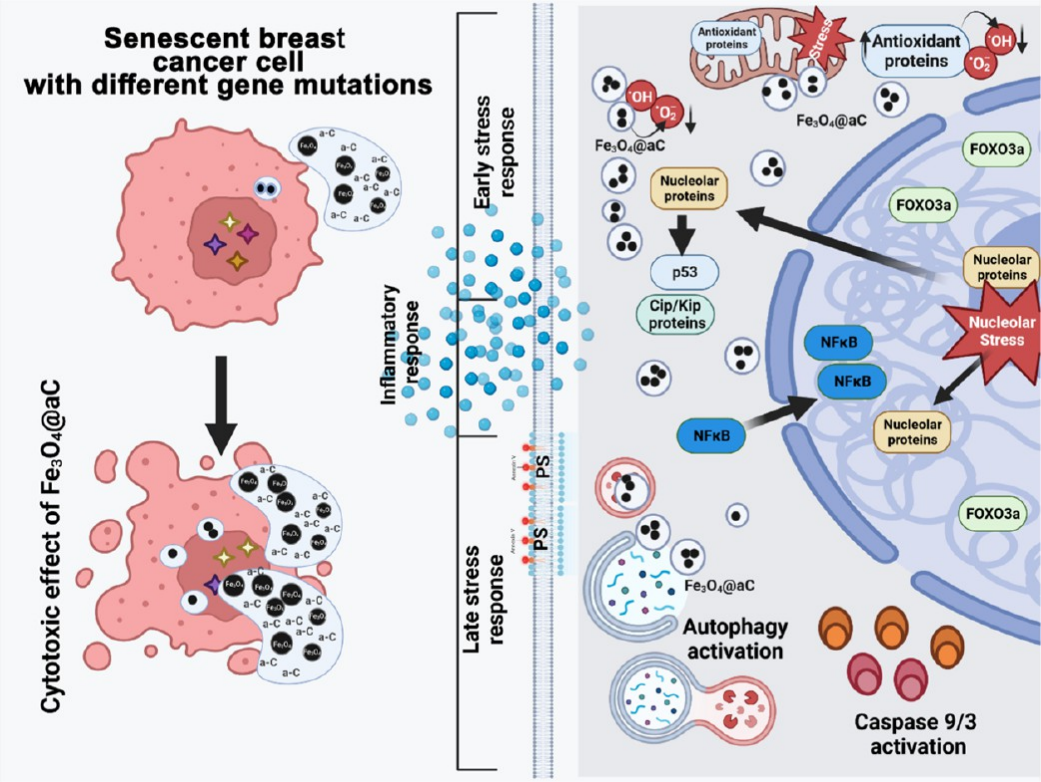
Carbon-coated iron oxide NPs (Fe_3_O_4_@aC) promote cytotoxic effects in drug-induced senescent breast cancer cells with different gene mutation statuses (left) that is mechanistically achieved by the induction of reductive stress (decreased ROS production, elevated levels of antioxidant proteins such as FOXO3a, SOD1, and GPX4) (right). Reductive stress-mediated cytotoxicity (right) was accompanied by inflammatory response (NFκB activation, increased secretion of IL-6 and IL-8), nucleolar stress (relocation of nucleolar proteins), increased levels of cell cycle inhibitors, and autophagy induction (increased levels of BECN1 and LC3B) that, in turn, led to apoptotic cell death as judged by phosphatidylserine (PS) externalization and caspase 9 (mitochondrial pathway of apoptosis) and caspase 3 activation. Created with BioRender.com.

We also showed, for the first time, that carbon-coated iron oxide nanoparticles might promote reductive stress (reduced levels of ROS, increased activity of FOXO3a and elevated levels of antioxidant enzymes), which, in turn, results in inflammatory response (the activation of NFκB and elevated production of IL-6 and IL-8), nucleolar stress (relocation of nucleolar proteins), cytotoxic autophagy (increased levels of BECN1 and LC3B), and finally apoptotic cell death in drug-induced senescent breast cancer cells (Figure 11). We propose that reductive stress-associated cytotoxicity upon stimulation with encapsulated iron oxide NPs with reductive activity may be considered as a novel antibreast cancer strategy; however, more studies are needed to document in more detail the underlining mechanism(s) and related responses in different types of cancer cells treated with redox-active nanomaterials.

## Data Availability

The data presented in this study are available in the Supporting Information.

## Supporting Information Available

Physicochemical characterization of Fe_3_O_4_@Dex and Fe_3_O_4_@aC NPs; TGA curves; results of DLS measurements; stability measurements; effects of Fe_3_O_4_@Dex and Fe_3_O_4_@aC on metabolic activity in breast cancer cells; effects of Fe_3_O_4_@Dex and Fe_3_O_4_@aC on metabolic activity and apoptosis induction in epithelial cell line MCF 10F and BJ fibroblasts, description: scavenging activity tests in cell-free system; results of the scavenging tests of Fe_3_O_4_@aC NPs; and comparison of FTIR spectra of Fe_3_O_4_@aC and Fe_3_O_4_@Dex with spectra of samples after the reaction with ROS (docx) List of the mutated gene set in eight breast cancer cell lines (genes related to cell cycle, stress, and autophagy) (xlsx) (ZIP)
