# Supplementary material for: Carbon-Coated Iron Oxide Nanoparticles Promote Reductive Stress-Mediated Cytotoxic Autophagy in Drug-Induced Senescent Breast Cancer Cells
Source: ACS Appl Mater Interfaces. 2024 Mar 14;16(12):15457–78. doi: 10.1021/acsami.3c17418 (PMC10982943; doi:10.1021/acsami.3c17418)
Supplement: Supplementary file 1 — am3c17418_si_001.zip [file am3c17418_si_001.zip › Supporting Information/Supporting Information.docx]

Supporting Information

Carbon-coated iron oxide nanoparticles promote reductive stress-mediated cytotoxic autophagy in drug-induced senescent breast cancer cells

*Anna Lewińska^a,1^, Adrian Radoń^b,1,*^, Kacper Gil^a^, Dominika Błoniarz^a^, Agnieszka Ciuraszkiewicz^b^, Jerzy Kubacki^c^, Mariola Kądziołka-Gaweł^c^, Dariusz Łukowiec^d^, Piotr Gębara^e^, Agnieszka Krogul-Sobczak^f^, Piotr Piotrowski^f^, Oktawia Fijałkowska^a^, Sylwia Wybraniec^a^, Tomasz Szmatoła^g^, Aleksandra Kolano-Burian^b^, Maciej Wnuk^a,*^*

^a^Institute of Biotechnology, College of Natural Sciences, University of Rzeszow, Pigonia 1, 35-310 Rzeszow, Poland

^b^Łukasiewicz Research Network - Institute of Non-Ferrous Metals, Sowińskiego 5, 44-100 Gliwice, Poland

^c^Institute of Physics, Faculty of Science and Technology, University of Silesia in Katowice, 75 Pułku Piechoty 1, 41-500 Chorzów, Poland

^d^Faculty of Mechanical Engineering, Silesian University of Technology, Konarskiego 18A, 44-100 Gliwice, Poland

^e^Department of Physics, Częstochowa University of Technology, Armii Krajowej 19, 42-200 Częstochowa, Poland

^f^Faculty of Chemistry, University of Warsaw, Pasteura 1, 02-093 Warsaw, Poland

^g^Center of Experimental and Innovative Medicine, University of Agriculture in Krakow, Mickiewicza 24/28, 30-059 Krakow, Poland

^1^These authors contributed equally to this work.

(^*^) **Corresponding authors:**

*adrian.radon@imn.lukasiewicz.gov.pl*

*mwnuk@ur.edu.pl*


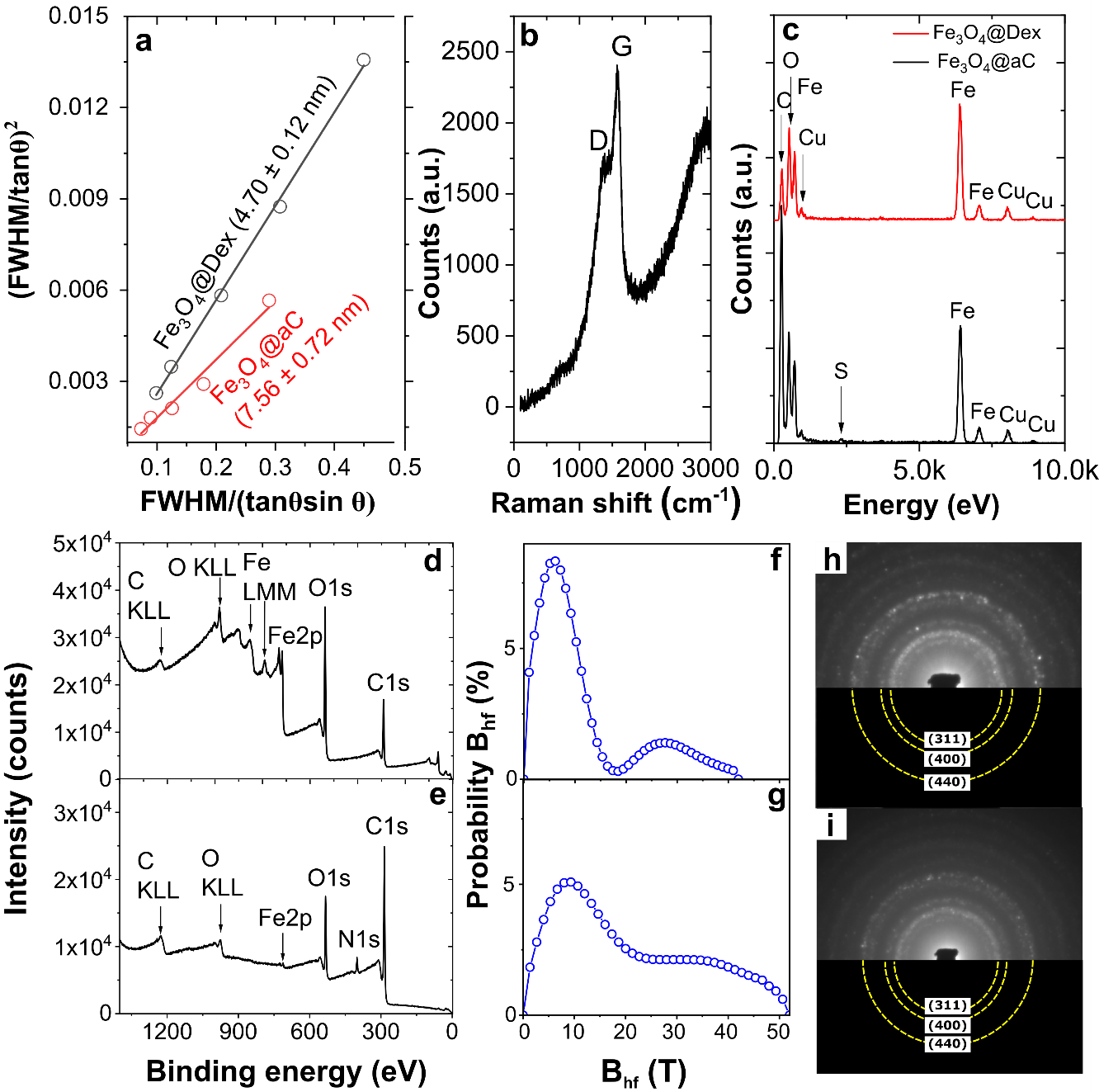


**Figure S1.** (**a**) Halder-Wagner plots of Fe_3_O_4_@Dex and Fe_3_O_4_@aC NPs with specified average crystallite size, (**b**) Raman spectrum of synthesized nanomaterial with marked bands characteristic for amorphous carbon presented in Fe_3_O_4_@aC, (**c**) EDX spectra recorded in STEM mode with marked identified chemical elements (presence of the Cu is associated with the sample preparation and using copper grid), (**d** and **e)** XPS survey spectra of Fe_3_O_4_@Dex (**d**) and Fe_3_O_4_@aC (**e**), (**f** and **g**) distribution function of effective magnetic fields for iron nuclei recorded for Fe_3_O_4_@Dex (**f**) and Fe_3_O_4_@aC (**g**); (**h** and **i**) indexed SAED ring patterns of Fe_3_O_4_@Dex (**h**) and Fe_3_O_4_@aC (**i**).


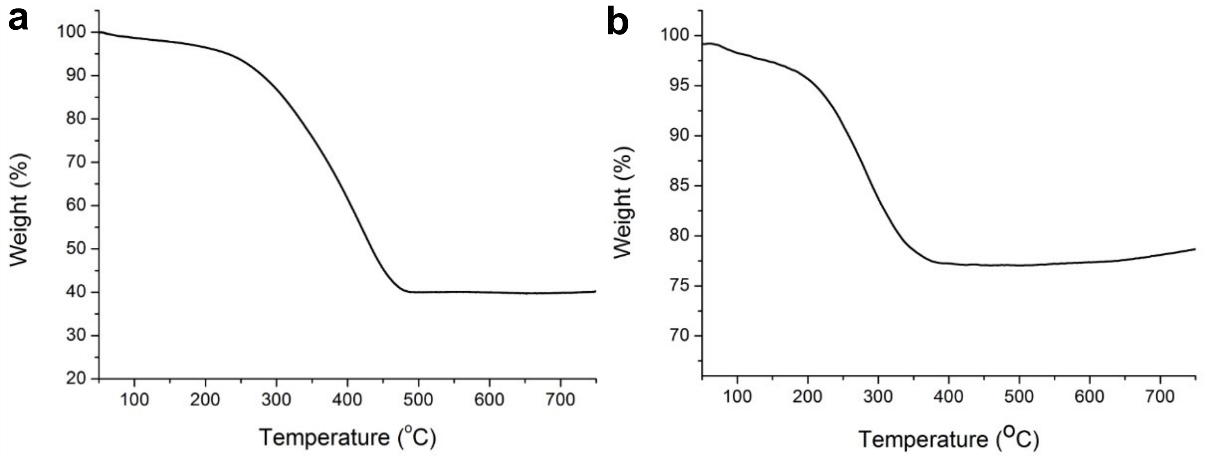


**Figure S2.** TGA curves recorded for (**a**) Fe_3_O_4_@aC and (**b**) Fe_3_O_4_@Dex samples under nitrogen atmosphere with heating rate of 5 ^o^C/min.


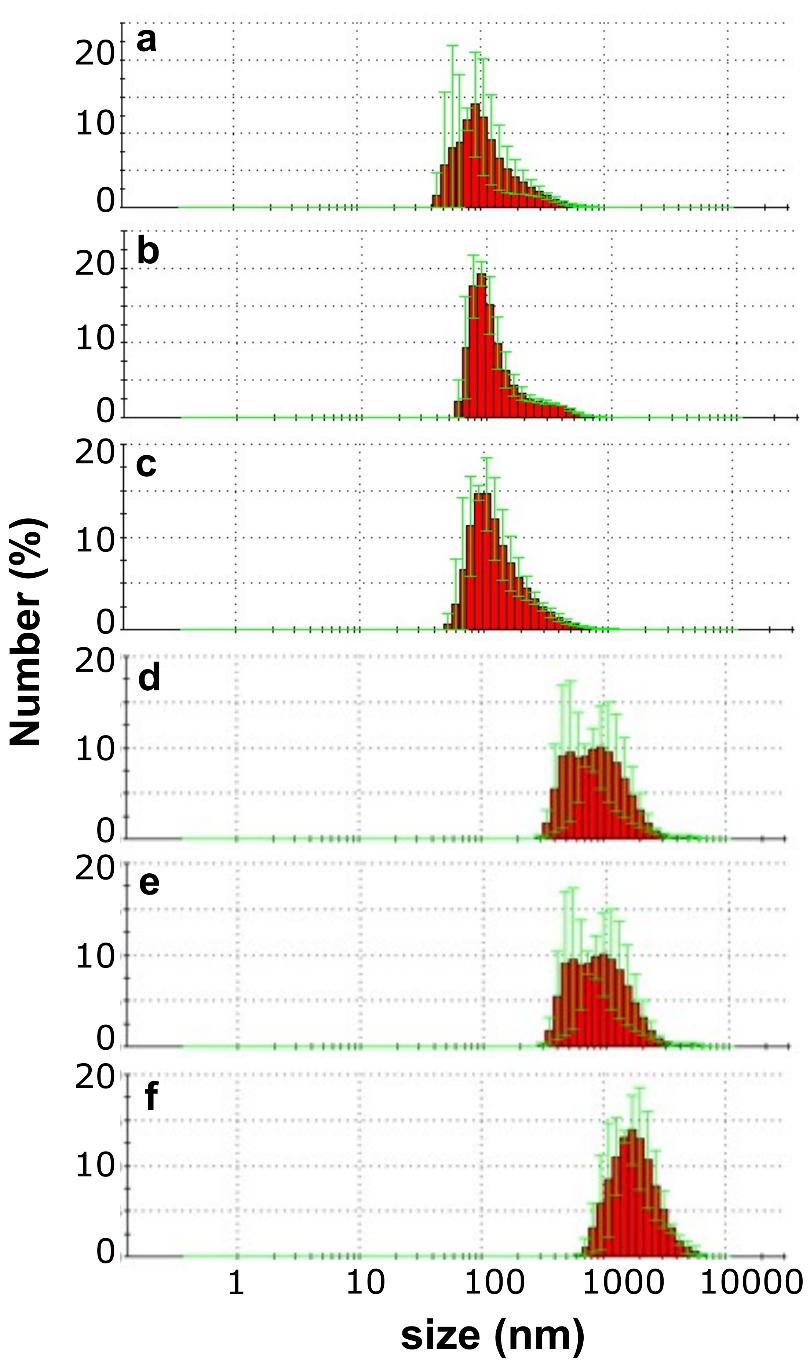


**Figure S3.** Results of DLS measurements for (**a**-**c**) Fe_3_O_4_@Dex and (**d**-**f**) Fe_3_O_4_@aC particles at the concentration of 1 mg/ml (**a** and **d**), 0.5 mg/ml (**b** and **e**) and 0.25 mg/ml (**c** and **f**).


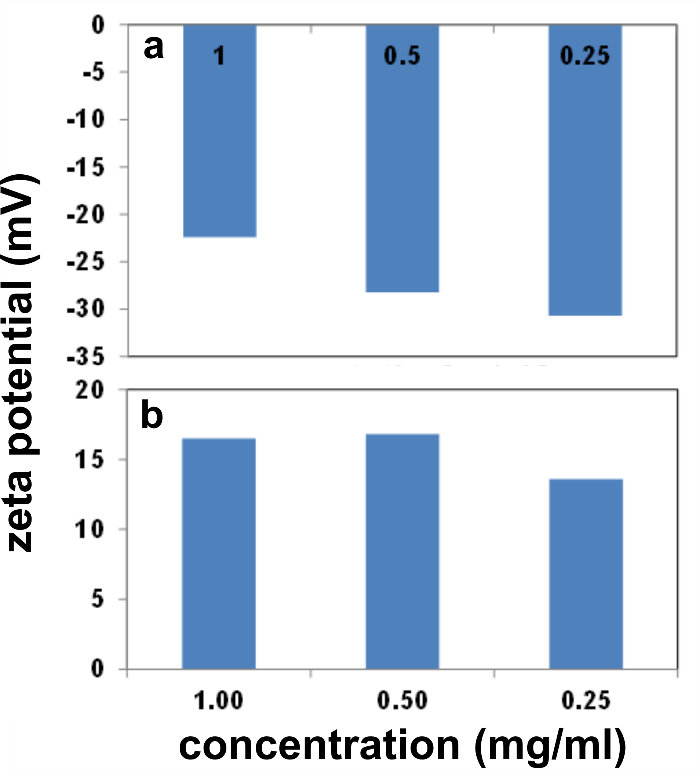


**Figure S4.** Stability measurements of (**a**) Fe_3_O_4_@Dex and (**b**) Fe_3_O_4_@aC particles using ζ potential depending on the concentration.


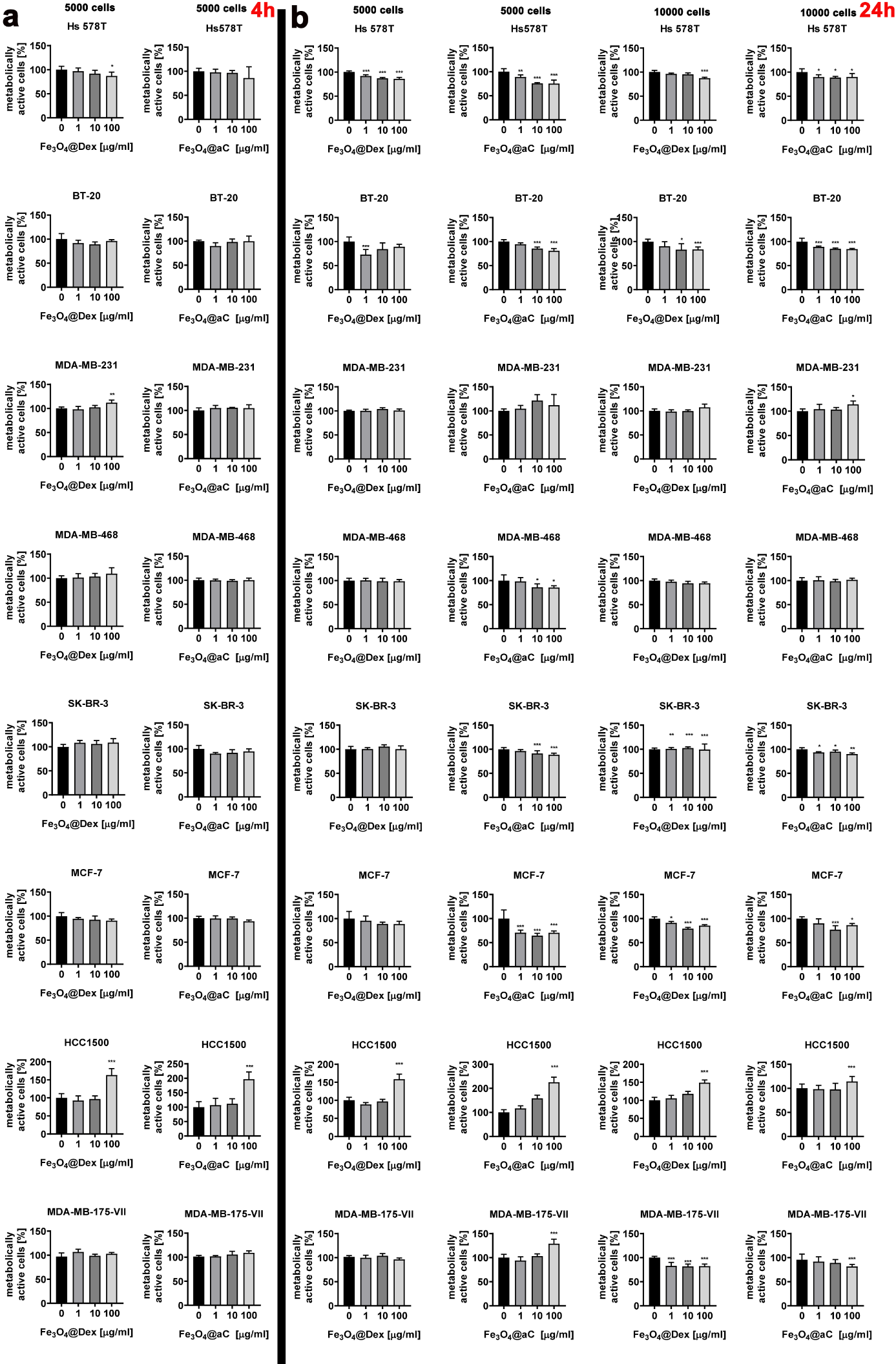


**Figure S5**. The effects of encapsulated Fe_3_O_4_ NPs (Fe_3_O_4_@Dex and Fe_3_O_4_@aC) on metabolic activity in breast cancer cells with different gene mutation status. Cells were treated with 1, 10 and 100 µg/ml NPs for 4 h (**a**) or 24 h (**b**). Metabolic activity was assayed using MTT test. Metabolic activity at standard growth conditions is considered as 100%. Bars indicate SD, n = 3, ^⁎⁎⁎^*p* < 0.001, ^⁎⁎^*p* < 0.01, ^⁎^*p* < 0.05 compared to untreated control (ANOVA and Dunnett’s a posteriori test). Fe_3_O_4_@Dex, dextran-based coated iron oxide nanoparticles; Fe_3_O_4_@aC, glucosamine-based amorphous carbon coated iron oxide nanoparticles.


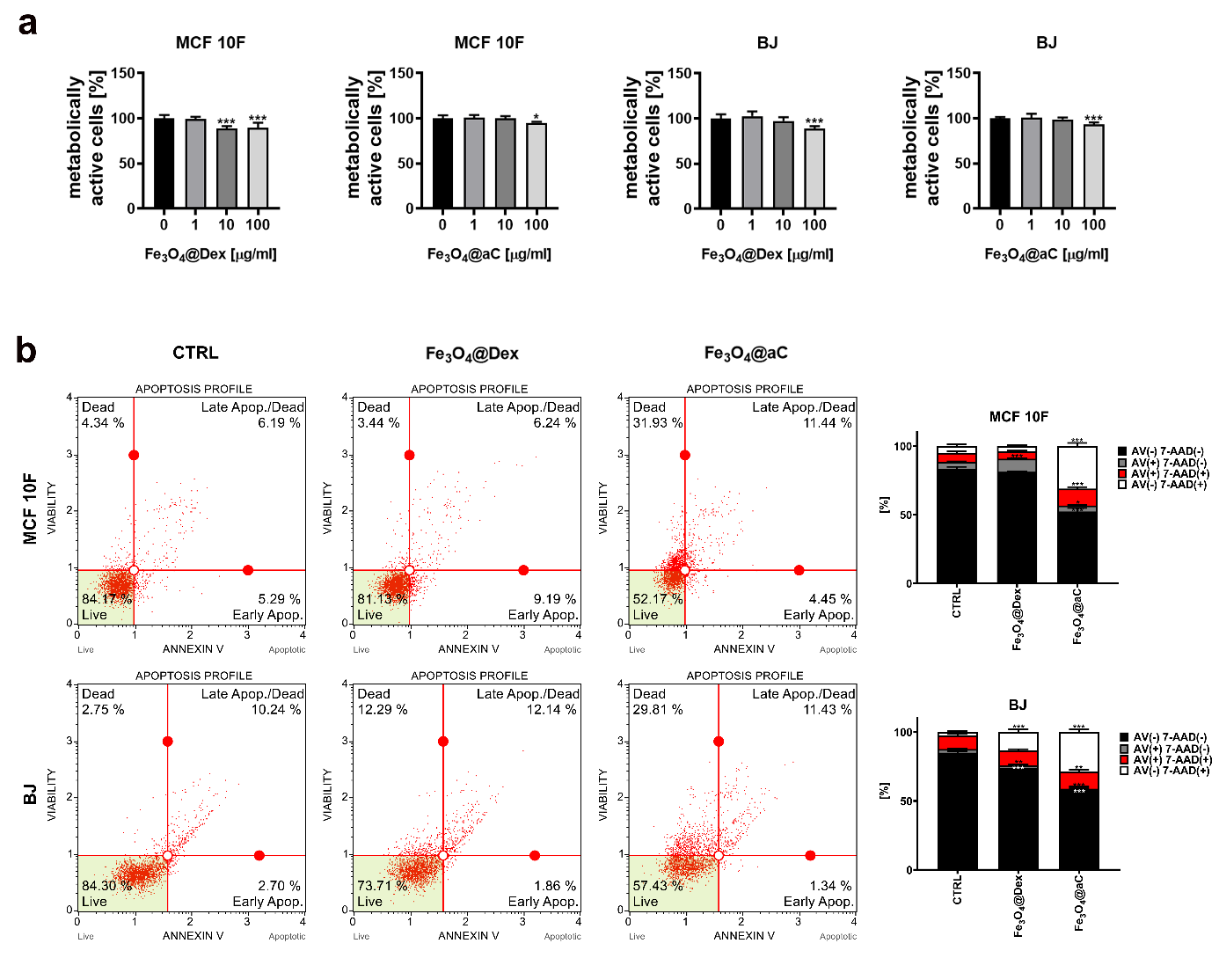


**Figure S6.** The effects of encapsulated Fe_3_O_4_ NPs (Fe_3_O_4_@Dex and Fe_3_O_4_@aC) on metabolic activity (**a**) and apoptosis induction (**b**) in normal human MCF 10F cells (a non-tumorigenic epithelial cell line) and BJ fibroblasts. Cells were treated with 1, 10 and 100 µg/ml NPs for 4 h (**a**) or with 100 µg/ml NPs for 4 h (**b**). (**a**) Metabolic activity was assayed using MTT test. Metabolic activity at standard growth conditions is considered as 100%. Bars indicate SD, n = 3, ^⁎⁎⁎^*p* < 0.001, ^⁎^*p* < 0.05 compared to untreated control (ANOVA and Dunnett’s a posteriori test). (**b**) Apoptosis and necrosis induced by encapsulated Fe_3_O_4_ NPs was revealed using Annexin V (phosphatidylserine externalization, an apoptotic marker) and 7-AAD (rupture of plasma membrane, a necrotic marker) dual staining and flow cytometry. Representative dot-plots are shown (**left**). (right) Bars indicate SD, n = 3, ^⁎⁎⁎^*p* < 0.001, ^⁎⁎^*p* < 0.01, ^⁎^*p* < 0.05 compared to untreated control (ANOVA and Dunnett’s a posteriori test). Four subpopulations are shown, namely live cells (Annexin V (AV)-negative, 7-AAD-negative), early apoptotic cells (Annexin V (AV)-positive, 7-AAD-negative), late apoptotic cells (Annexin V (AV)-positive, 7-AAD-positive) and necrotic cells (Annexin V (AV)-negative, 7-AAD-positive). CTRL, untreated control; Fe_3_O_4_@Dex, dextran-based coated iron oxide nanoparticles; Fe_3_O_4_@aC, glucosamine-based amorphous carbon coated iron oxide nanoparticles.

**Scavenging activity tests in cell-free system**

To determine the scavenging properties of Fe_3_O_4_@aC NPs, a set of tests based on the degradation of model organic dye – Rhodamine B (RhB) was performed. For all experiments, a water dispersion of the nanoparticles with a 6 mg/ml concentration was prepared using ultrasounds. The concentration of the RhB in the reaction solution was determined based on the UV spectra measurements. The measurements were performed in the same manner in all cases, i.e., 3 ml of reaction solution was taken and centrifugated at 10000 rpm to remove the nanoparticles. Afterward, the clear RhB solution was placed in the quartz cuvette, and the UV-Vis spectra in the 400 – 750 nm wavelength range were measured using UV-Vis spectrophotometer Evolution 220. The changes in the concentrations were then determined based on the prepared calibration curve.

Firstly, the adsorption properties were studied. For this purpose, the 2 ml of the prepared dispersion was magnetically stirred in the dark for 60 minutes with the 4 ml of the RhB water solution of an initial concentration of 0.025 mM. The adsorption efficiency (expressed as the % of the dye removal) and adsorption capacity were calculated using the final reaction concentration after the adsorption test and the initial concentration.

The hydrochar-like amorphous carbon shell formation increases the tested dye’s adsorption rate. While for the Fe_3_O_4_@Dex, the absorption capacity was equal to 1.24 mmol/g (89.94 % of RhB was absorbed in 1 hour), for the Fe_3_O_4_@aC, this parameter was equal to 1.36 mmol/g and 97.56% of RhB was adsorbed. This observation is associated with the differences in the NPs charge. The positive ζ potential observed for the Fe_3_O_4_@aC is related to the positively charged surface, while the Fe_3_O_4_@Dex opposite scenario can be observed. Generally, carboxyl and carbonyl groups are associated with the negative zeta potential, while the oxygen-free carbon sites can be protonated in the water solution, which is compatible with XPS results. In the pH of 4.4 (measured for the analyzed system), the RhB is presented in zwitterionic form. Therefore, the positively charged surface of Fe_3_O_4_@aC is responsible for the strong electrostatic interaction with the COO^¯^. In the case of the Fe_3_O_4_@Dex, the surface is negatively charged, so this interaction can also appear relating to the zwitterionic state of RhB.

The scavenging properties of the Fe_3_O_4_@aC NPs were determined in the UV/H_2_O_2_ system, in which the ROS degrades RhB (**Fig. S7**). Firstly, the degradation efficiency was tested for the system without adding nanoparticles. For this purpose, 8 ml of RhB solution was diluted using 4 ml water, and 1 ml of H_2_O_2_ (30%) was added. The reaction was performed using 365 nm UV light, and the RhB concentration was determined based on the UV-Vis spectra analysis recorded after 0, 15, 30, 45, and 60 minutes. Similarly, the second experiment was performed using NPs. The 0.5 ml of DI water was replaced by the Fe_3_O_4_@aC NPs dispersion, and the reaction process was preceded by the adsorption of the RhB on the nanostructure. For this purpose, the reaction solution (without the H_2_O_2_ addition) was stirred in the dark for 60 min, and the initial concentration of RhB in the solution was determined after this time needed to obtain sorption-desorption equilibrium. Afterward, the hydrogen peroxide was added, and the reaction was performed similarly to the system without nanoparticles.

To determine the changes in the structure of amorphous-like carbon shells in Fe_3_O_4_@aC NPs and on the surface of Fe_3_O_4_@Dex NPs with mild reduction properties, the FTIR spectra were measured before and after the exposure of the nanoparticles to the HO^●^ radicals (**Fig. S8**). For this purpose, 10 ml of the nanoparticles dispersion was diluted using 10 ml DI water, and 2 ml of H_2_O_2_ (30%) was added. The generation of the hydroxyl radicals was then initialized using 254 nm UV light, and the reaction was conducted for 5 h to ensure the complete structural changes on the nanoparticles’ surface. Afterward, the nanoparticles were collected using centrifugation (10000 rpm/5 min), washed thrice with DI water, and dried at 70 ℃ for 12 h. The FTIR spectra were collected using the KBr pellet method.





**Figure S7.** The results of the scavenging tests of Fe_3_O_4_@aC NPs: (**a**) differences between degradation efficiency in the UV/H_2_O_2_ system with and without the addition of Fe_3_O_4_@aC NPs; (**b**) and (**c**) UV-Vis spectra recorded for the UV/H_2_O_2_ and UV/H_2_O_2_/ Fe_3_O_4_@aC systems, respectively.





**Figure S8.** Comparison of FTIR spectra of Fe_3_O_4_@aC (**a**) and Fe_3_O_4_@Dex (**b**) with spectra of samples after the reaction with ROS ((OH)-marked samples); on spectra marked also identified, characteristic functional groups formed after this reaction.

TABLE OF CONTENTS (TOC)

| **Description of Supporting Information** | **page** |
| --- | --- |
| Supporting Information – title page | 1-2 |
| **Figure S1.** Physico-chemical characterization of Fe_3_O_4_@Dex and Fe_3_O_4_@aC NPs. | 3 |
| **Figure S2.** TGA curves. | 4 |
| **Figure S3.** Results of DLS measurements. | 5 |
| **Figure S4.** Stability measurements. | 6 |
| **Figure S5.** The effects of Fe_3_O_4_@Dex and Fe_3_O_4_@aC on metabolic activity in breast cancer cells. | 7-8 |
| **Figure S6.** The effects of Fe_3_O_4_@Dex and Fe_3_O_4_@aC on metabolic activity and apoptosis induction in epithelial cell line MCF 10F and BJ fibroblasts. | 9 |
| Description: Scavenging activity tests in cell-free system | 10-11 |
| **Figure S7.** The results of the scavenging tests of Fe_3_O_4_@aC NPs. | 12 |
| **Figure S8.** Comparison of FTIR spectra of Fe_3_O_4_@aC and Fe_3_O_4_@Dex with spectra of samples after the reaction with ROS. | 13 |
| Table Of Contents (TOC) | 14 |
